# Towards a supervised classification of neocortical interneuron morphologies

**DOI:** 10.1186/s12859-018-2470-1

**Published:** 2018-12-17

**Authors:** Bojan Mihaljević, Pedro Larrañaga, Ruth Benavides-Piccione, Sean Hill, Javier DeFelipe, Concha Bielza

**Affiliations:** 1 Departamento de Inteligencia Artificial, Universidad Politécnica de Madrid, Boadilla del Monte, 28660 Spain; 2 Laboratorio Cajal de Circuitos Corticales, Universidad Politécnica de Madrid and Instituto Cajal (CSIC), Pozuelo de Alarcón, 28223 Spain; 3 Krembil Centre for Neuroinformatics, Centre for Addiction and Mental Health, Toronto, M5T 1R8 Canada; 4 Blue Brain Project, École Polytechnique Fédérale de Lausanne, Genève, CH-1202 Switzerland

**Keywords:** Feature selection, Martinotti, Morphometrics

## Abstract

**Background:**

The challenge of classifying cortical interneurons is yet to be solved. Data-driven classification into established morphological types may provide insight and practical value.

**Results:**

We trained models using 217 high-quality morphologies of rat somatosensory neocortex interneurons reconstructed by a single laboratory and pre-classified into eight types. We quantified 103 axonal and dendritic morphometrics, including novel ones that capture features such as arbor orientation, extent in layer one, and dendritic polarity. We trained a one-versus-rest classifier for each type, combining well-known supervised classification algorithms with feature selection and over- and under-sampling. We accurately classified the nest basket, Martinotti, and basket cell types with the Martinotti model outperforming 39 out of 42 leading neuroscientists. We had moderate accuracy for the double bouquet, small and large basket types, and limited accuracy for the chandelier and bitufted types. We characterized the types with interpretable models or with up to ten morphometrics.

**Conclusion:**

Except for large basket, 50 high-quality reconstructions sufficed to learn an accurate model of a type. Improving these models may require quantifying complex arborization patterns and finding correlates of bouton-related features. Our study brings attention to practical aspects important for neuron classification and is readily reproducible, with all code and data available online.

**Electronic supplementary material:**

The online version of this article (10.1186/s12859-018-2470-1) contains supplementary material, which is available to authorized users.

## Background

Although GABAergic interneurons constitute only 10–30% of the neurons in the neocortex they are highly diverse with regards to morphological, electro-physiological, molecular, and synaptic properties [1–8]. Most researchers consider that interneurons can be grouped into types [9] with much less variability within types than among them. High-throughput generation of data is expected to enable learning a systematic taxonomy within a decade [10], by clustering [11, 12] molecular, morphological, and electrophysiological features. Currently, however, researchers use (e.g., [13],) and refer to established morphological types such as chandelier (ChC), Martinotti (MC), neurogliaform (NGC), and basket (BA) [6, 8, 14, 15]. These types are identified on the basis of the target innervation location —e.g., the peri-somatic area for basket cells— and somatodendritic and axonal morphological features. The latter can be subjective and lead to different classifications: e.g., while [16] distinguish between large, nest, and small basket cell types, based on features such as axonal arbor density and branch length, [14] only distinguish between large and common basket types. There is thus no single catalogue of types, and the different classification schemes [6, 14] only partially overlap. There is, however, consensus on the morphological features of the ChC, MC, and NGC types [14].

Using a trained model to automatically classify interneurons into these morphological types [17] could bring insight and be useful to practitioners [14]. A sufficiently simple and accurate model would provide an interpretable mapping from the quantitative characteristics to the types, such as, for example, the classification tree [18] model by [19] relating mRNA expression to anatomical type. Unlike classification by an expert, a classifier’s assignment of an interneuron into a particular type can be understood by analyzing the model, and many models can quantify the confidence in their decision. Identifying cells that the model cannot reliably classify into any of the a priori known types might lead to refining the classification taxonomy, as these cells might belong to a novel type, or suggest that the boundary between a pair of types is unclear if the model finds many interneurons very likely to belong to either type. Sufficiently accurate models could be used by all practitioners to ‘objectively’ classify interneurons, rather than each of them assigning their own classification. Learning such models may help enable future unsupervised type discovery by identifying and fostering the development and definition of useful morphometrics. Such models can be trained in a supervised fashion [20–22], with the cells pre-classified (labeled) into a number of a priori specified types. With thousands of neuronal morphology reconstructions [23, 24] available at online repositories such as Neuromorpho.org [25, 26] and the Allen Brain Cell Types Database^1^, this seems more attainable than ever, especially for the rodent brain.

There are, however, practical obstacles and aspects to consider when learning such models. First, it is important that class labels (i.e., the a priori classification) are assigned according to well-established criteria, to avoid learning idiosyncrasies of the annotating neuroscientist. Second, reconstructions at Neuromorpho.org are often incomplete (e.g., insufficient axonal length or interrupted axons), lack relevant metadata, such as the cell body’s cortical area and layer, and there is a lot of variability if combining data across species, age, brain region [4], as well as histological, imaging, and reconstruction protocol [27–29], whereas focusing on a homogeneous data set shrinks the sample size. Third, infinitely many morphometrics [30] —variables that quantify morphological features— can be computed and their choice will influence the model [31]. While the Petilla convention [9] provided a reference point by identifying a set of features to distinguish interneuron types, only some of them are readily quantified with software such as L-Measure [32] and Neurolucida Explorer (MicroBrightField), as many either rely on often-missing metadata (e.g., laminar extent), or are vaguely defined (e.g., ‘dense plexus of highly branched axons’). Indeed, researchers have often resorted to quantifying interneurons with custom-computed morphometrics [13, 33–35].

In the present study we learned models from 217 high-quality reconstructions, namely two-week-old male rat hind-limb somatosensory cortex interneurons, reconstructed at the Laboratory for Neural Microcircuitry at the École Polytechnique Fédérale de Lausanne [36]. Each cell was pre-classified into one of eight morphological types described in [6]^2^. With only seven ChC and 15 bitufted (BTC) —yet as many as 123 BA and 50 MC— cells, the sample was insufficient to accurately distinguish each of the eight types, yet the homogeneity and quality of the data, along with a careful selection of morphometrics and a comprehensive machine learning approach, allows for establishing a baseline classification. Although the class labels were assigned following clear criteria, they came from a single laboratory, and we thus contrasted them (for 20 cells) with alternative labels provided by 42 leading neuroscientists that participated in [14]. We also looked for morphology reconstruction issues which might distort the morphometrics. We trained a model for each type in a one-versus-all fashion (e.g., ChC or not ChC; see [37],). Importantly, we developed custom R [38] code to quantify a number of Petilla features, including those regarding: arbor shape and direction; dendritic polarity; the presence of arborization patterns typical of the MC and ChC types; and translaminar extent [34], which we estimated using metadata on laminar thickness and soma’s laminar location (i.e., which layer contained the soma). We complemented them with standard axonal and dendritic morphometrics [30], such as the mean branching angle and mean terminal branch length, computed with the NeuroSTR library^3^. For each classification task (e.g., ChC or non-ChC), we ran nine well-known supervised classification algorithms [20, 21], such as random forest ([39],) and lasso-regularized logistic regression [40]. As a prior step, we applied univariate and multivariate feature selection [41, 42] and sampled the training data to deal with class imbalance (e.g., there were seven ChC and 210 non ChC cells; see [43, 44],). We validated the MC models against the classification by 42 neuroscientists from [14] and illustrated how cells commonly misclassified by different models [45] may correspond to atypical MC morphologies^4^. The study can be easily reproduced [46–48] as all code and data are available^5^.

### Morphological classification

**Fig. 1:**
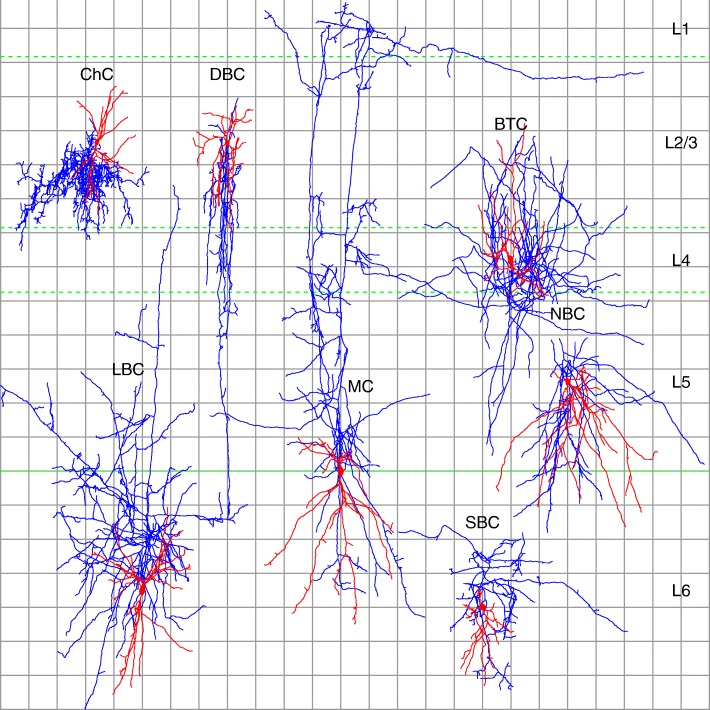
Examples of the eight morphological types from [6] for which we learned supervised models. The types are: bitufted (BTC); chandelier (ChC); double bouquet (DBC); large basket (LBC); Martinotti (MC); nest basket (NBC); small basket (SBC), and the compound basket (BA) type, composed of NBC, LBC, and SBC cells. Neurogliaform (NGC) and bipolar (BP) types not shown as we omitted them from supervised classification, because we had only three cells of each. Typical features, according to [6], include: bitufted dendrites (BTC); sharply branching axons and low bouton density (LBC); and axons with spiny boutons, reaching L1 (MC); and vertical rows of boutons (ChC). Axons are drawn in blue with dendrites and somata in red. Dashed green lines indicate layer boundaries from the rat hind-limb somatosensory cortex. There are 100 *μm* between consecutive grid lines

Since the early studies of Santiago Ramón y Cajal it has generally been assumed that interneurons belong to distinct classes [2, 49–51]. There is, however, no universally accepted catalog of such classes [9, 14]. [6] provided a widely cited morphological classification scheme for inhibitory interneurons in layers L2/3 to L6. It specifies nine distinct types (see Fig. 1 for a listing and acronym definitions) on the basis of axonal and dendritic features, including fine-grained ones such as bouton distribution. This scheme is often refined (e.g., [7, 13],) by adding a layer prefix to each type (e.g., L23_MC, L4_MC, etc.) for a total of 4×9=36 types. [14] proposed an alternative, pragmatic classification scheme, based only on high-level patterns of axonal and dendritic arborization. It partially overlaps with the [6] scheme, sharing the NGC, ChC, and MC types^6^. In [14] 42 leading neuroscientists classified a set of interneurons by looking at 2D and 3D morphology images (they also knew the layer containing the soma) and found that the ChC and, to a lesser degree, MC and NGC types could be identified from high-level morphology alone, as the neuroscientists largely agreed when deciding whether or not a cell was a member of these types.

### Digital reconstructions

A typical neuronal morphology reconstruction [23] is a sequence of connected conical frusta [52], called segments (or compartments), each characterized by six values: the Euclidean coordinates (X, Y and Z) and radius of its terminating point, all given in *μm*; the identity of its parent segment; and its process type (soma, dendrite or axon); with the soma’s centroid usually at coordinates (0,0,0). A branch is the sequence of segments between two bifurcation points (i.e., terminal point of a segment having multiple child segments), while linked branches form an arbor. The reconstructions are most commonly traced by hand [23] and there is substantial inter-operator variability [27], especially regarding fine-grained properties, such as dendritic and axonal thickness and local branching angles, while bouton locations are seldom included. In addition, histological processing of brain slices makes the tissue shrink, increasing arbor tortuosity (decreasing reach while maintaining total length) [53]. Current efforts to improve and standardize automatic reconstruction, such as BigNeuron [29] may remove reconstruction-specific differences, increasing the usability of morphologies produced.

### Morphometrics

The Petilla convention [9] established a set of morphological features that distinguish cortical interneuron types. They include characteristics such as: branching angles; axon terminal branch shape (curved / straight); bouton density and clustering patterns; dendritic polarity; whether the axon is ascending or descending; whether it is intra- or trans-laminar; or presents distinctive patterns of arborization, such as ‘bundles of long, vertical branches or tufts’ or ‘dense plexus of highly branched axons’. Many of these correspond to standard neuronal morphometrics (e.g., branching angles) or can be quantified rather directly (e.g., one can compute the tortuosity of terminal branches). Others either a) are often impossible to quantify, since relevant data (e.g., bouton density) may be missing from the digital morphology reconstruction; b) can only be approximated (e.g., translaminar extent) as the data is often incomplete (we often only know the soma’s layer, not the position of the soma within the layer); or c) are vaguely defined (e.g., ‘dense plexus of highly branched axons’).

Standard neuronal morphometrics [30] are either metric (e.g., branch length) or topological (partition asymmetry; [54],), and are computed either at the whole arbor(s) level (e.g., height) or for a part of the tree, such as a branch or a bifurcation (e.g., branch length); the latter are then quantified with summarizing statistics across the arbor(s) (e.g., mean and maximal branch length). These morphometrics can be computed with software such as the free L-Measure [32], the commercial Neurolucida Explorer (MicroBrightField), and open-source alternatives being actively developed such as NeuroSTR and NeuroM^7^. L-measure provides 42 analyses of morphology, with five summary statistics per analysis; 19 out of the 42 analyses depend on arbor diameter or local bifurcation angles, which often differ across laboratories [27, 28], and it seems to assume bifurcating branches, although multifurcations can occur [55].

Researchers have often quantified interneurons with custom-implemented morphometrics such as: the mean X coordinate of the axon (e.g., [13],); 2D (X and Y) axonal ‘tile surface’ and density [35]; the extent of axonal arborization in L1 [34]; features derived from 2D axonal and dendritic density maps [7]; dendritic polarity [33]; estimates of translaminar extent and of the radial (ascending or descending) direction of arborization [56]; or the position of the convex hull’s centroid as a proxy for arbor orientation and extent [35, 56].

## Method

Here we provide an overview of the applied methodology. Details, such as the definitions of morphometrics, are provided in Additional file 1.

### Data

We used 228 hind-limb somatosensory cortex interneuron morphologies from two-week-old male Wistar (Han) rats. These cells were previously reconstructed by the Laboratory for Neural Microcircuitry and then used by [13] for simulating a cortical microcircuit^8^. They corrected shrinkage along the Z-axis, while shrinkage along the X and Y axes was of approximately 10%. They classified the cells into 36 layer L2/3 to layer L6 morphological types of inhibitory neurons, based on their soma’s layer and anatomical features described in [6, 16, 57], updating these criteria with a few laminar specificities: e.g., L6 MC cells were unique in that they did not reach L1, but ‘had a second axonal cluster formed below L1’ ([13],page 2 in the supplementary material). For each cell, we knew which layer contained the soma and had estimates of mean and standard deviation of cortical layers’ thickness (see Table S3 in the Additional file 1). We had no data on fine-grained features related to boutons and dendritic spines. We merged the interneuron types across layers (e.g., we considered L23_MC and L4_MC cells as members of a single MC class) into the nine morphological types defined by [6].

We had an alternative classification for 79 of our cells provided by 42 neuroscientists that participated in the study by [14], who were shown 2D and 3D images of the cells and were told the layer containing the soma, and classified them following the scheme by [14]. Among these, we used the 20 cells^9^ classified in our data —that is, by [13]— as MC, ChC, and NGC —the three types common to both classification schemes— to contrast the neuroscientists’ labels to ours, but we did not use them to train the models. We will reserve the term ‘our labels’ to the labels by [13] which we trained the models with.

**Fig. 2:**
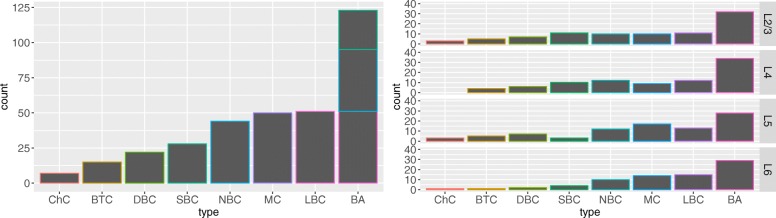
Frequencies of interneuron types in our data: overall (left) and per cortical layer (right). This figure shows the 217 cells used for supervised classification, with the SBC, NBC, and LBC types also shown in the bar corresponding to BA (i.e., the BA bar does not contribute to total cell count)

For supervised classification, we omitted the BP and NGC types, as we had only three examples of each and formed a compound type —basket (BA)— by merging the NBC, LBC, and SBC cells. We also omitted five cells with morphology issues: three cells whose axonal arborization was interrupted, and two with short axons (2500 *μm* and 2850 *μm*)^10^, thus obtaining the final sample of 217 cells from eight interneuron types (seven ‘base’ types plus the compound BA type) used for supervised classification (see Fig. 2).^11^

### Morphometrics

**Fig. 3:**
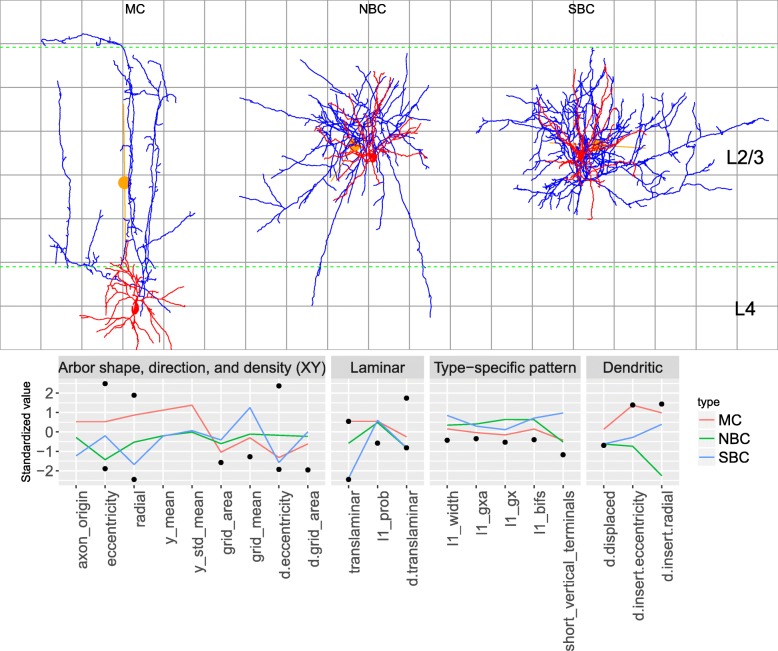
Custom-implemented morphometrics for an L4 MC (top panel: left; bottom panel: red), an L2/3 NBC (top: middle; bottom: green), and an L2/3 SBC (top: right; bottom: blue) interneuron. The bottom panel shows standardized values, with black dots indicating minima and maxima (extrema outside (−2.5,2.5) not shown). The axon of the MC cell originates from the upper part of the soma (axon_origin), grows along a radial axis (eccentricity, radial; axis drawn with the orange line), radially far from the soma (y_mean, center of mass shown with orange dot) and above it (y_std_mean), covers a small surface (grid_area), and its branches are not clustered together (grid_mean). It is translaminar (translaminar) and there is just a moderate (around 30%) probability of it reaching L1 (l1_prob) because, even with its soma vertically in the middle of L4, it only touches the bottom of L1. Low l1_prob and arbor width produce a low estimate of width (l1_width), bifurcations count (l1_bifs), and horizontal fanning out (l1_gxa) in L1. The dendritic arbor of the MC cell is displaced (d.displaced) from the axon and the dendrites stem from opposite ends of the soma (d.insert.eccentricity), located along a radial axis (d.insert.radial). The NBC cell’s axonal arbor is circular (radial), with closely grouped branches (grid_mean)) and a number of short vertical terminals (short_vertical_terminals). The axon of the SBC cell is intralaminar, tangentially oriented, with closely grouped branches, while both cells’ dendrites are spread out (multipolar) and colocalized with the axons. Dashed green lines indicate layer boundaries from the rat hind-limb somatosensory cortex, assuming that the somas are located in the middle of their layer. Axon is shown in blue with dendrites and somata in red. The grid lines are at 100 *μm* from each other. Dendritic morphometrics are prefixed with d.. Axon terminal branch morphometrics, not shown here, are prefixed in the remainder of the text with t

We computed a total of 103 axonal and dendritic morphometrics, 48 of which were custom-quantified Petilla [9] features. The custom-implemented morphometrics cover a) arbor shape, direction, density and size; b) laminar distribution; c) dendritic polarity and displacement from axonal arbor; and d) the presence of arborization patterns typical of the MC, ChC, and LBC types. We determined arbor orientation with principal component analysis, following [58]. We quantified laminar distribution as the probability of the arbor reaching at least two layers (one being its soma’s home layer), given that the soma’s vertical position within its layer was unknown and that laminar thicknesses were random variables rather than precise values. We distinguished between bipolar/bitufted and multipolar dendrites by determining whether dendrite roots were located along a single axis (for an alternative metric see [33]). Finally, we quantified a number of complex, type-specific patterns with simple, ad-hoc morphometrics. For the MC type, we quantified the ‘axonal collaterals that reach layer L1 and then ramify to form a fan-like spread of axonal collaterals’ [9] pattern by considering the estimated probability of the axon reaching L1, together with properties, such as width, of the upper part of the arbor. For ChC, we counted the number of ‘short vertical terminal branches’. We did not estimate translaminar extent as, without knowing the soma’s location within the column, it is poorly correlated to tangential arborization span [34]. Figure 3 illustrates some of these morphometrics.

The remaining 55 morphometrics were standard metric and topological [30] ones, such as bifurcation angles and partition asymmetry [54], including features of axon terminal branches such as length and curvature. We avoided morphometrics that are possibly sensitive to reconstruction granularity, such as those derived from axonal and dendritic diameter, local bifurcation angles, or segment length (e.g., the Fragmentation and Length analyses in L-Measure), as we had two groups of cells that differed sharply in terms of mean diameter and segment length.

We computed the morphometrics with the open-source NeuroSTR library and custom R [38] code. NeuroSTR allowed us to handle multifurcations (e.g., we ignored angle measurements on multifurcating nodes) and compute arbitrary statistics, so that, for example, we were able to compute the median branch length. Still, a number of potentially useful morphometrics available in Neurolucida Explorer, such as box counting fractal dimension [59], were not available in NeuroSTR and thus were not considered in this study. Additional file 1 (Section 1) lists all the morphometrics used, with definitions and computation details.

### Supervised classification

Rather than training models to distinguish among all interneuron classes at once, we considered eight settings where we discerned one class from all the others merged together (e.g., whether a cell is a ChC or a non-ChC cell). One benefit of this is that we can interpret such models, and look for relevant morphometrics, in terms of that particular type. On the other hand, training these models suffers from class imbalance ([43],); this was most pronounced for the ChC type (there were seven ChC cells and 210 non ChC cells), and least pronounced for BA (123 BA and 94 non-BA cells), which was the only setting in which the class of interest was the majority one (i.e., there were more BA than non-BA cells).

To each classification setting we applied nine supervised classification algorithms (see Table 1 for a list with abbreviations), such as random forest (RF), single-layer neural networks (NNET), and support vector machines (SVM), covering all main ‘families’ of classifiers. RF and SVM are among the most accurate classifiers available [60], while lasso regularized logistic regression (RMLR) and classification and regression trees (CART) can provide parsimonious and interpretable models.

**Table 1:** Classification algorithms and their parameterization

Classifier	Abbreviation	R Package	Prespecified Parameters
Classification and regression trees	CART	rpart [71]	$|\mathcal {D}^{a}|$ = 10, $|\mathcal {D}^{l}| = 5$
*k* nearest neighbors	kNN	kknn [72]	*k*=5,*p*=2 unweighted
Linear discriminant analysis	LDA	MASS [73]	
Gaussian naive Bayes	NB	e1071 [74]	
Random forest	RF	randomForest [75]	$T = 2000, m = \sqrt {n}$
Lasso regularized logistic regression	RMLR	glmnet [76]	*λ*=0.01
Support vector machine	SVM	e1071 [74, 77]	RBF: $\gamma = \frac {1}{n}, C = 1$
Single-layer neural network	NNET	neuralnet [78]	*h*=5
AdaBoost	ADA	gbm [79]	*T*=3000*d*=1*s*=0.001

For kNN, *p*=2 stands for Euclidean distance. RBF: radial basis function. Remaining parameters are defined in the Additional file 1/. R package is the library implementing the method

Briefly, NB approximates the joint probability distribution over the class and the features *P*(*c*,**x**) by assuming the features **x** are independent given the class *c*, while LDA assumes that each class-conditional density *p*(**x**∣*c*) is a multivariate Gaussian with a mean ***μ***_***c***_ and a covariance matrix ***Σ*** common to all classes. RMLR approximates *P*(*c*∣**x**) with a linear function of **x**, fitting its coefficients ***β*** by regularized maximum likelihood estimation. The ***β*** are interpretable: keeping all other features fixed, a unit increase in a standardized feature *X*_*j*_ increases the log-odds of the positive class by *β*_*j*_. NNET models *P*(*c*∣**x**) as a linear combination of derived features, each of which is in turn a linear combination of **x**. The SVM finds the maximal margin hyperplane that separates two classes while projecting the data onto a higher dimensional space. CART recursively partitions the training samples by considering a single feature at a time. RF and ADA are ensembles of *T* classification trees. RF learns *T* trees from *T* bootstrap samples of the training data, while ADA learns each tree in the sequence by giving more weight to instances misclassified by the previous tree. kNN classifies an instance **x** by choosing the most common class label among its *k* nearest neighbors in feature space.

We handled class imbalance with a hybrid of random undersampling and SMOTE oversampling (e.g., [61],), meaning that we removed (added) some majority (minority) class instances from (to) the training data. We also pruned the set of morphometrics [41] by keeping only those that were relevant according to the Kruskal-Wallis^12^ (KW) statistical test [62] and our adaptation of the RF variable importance (RF VI) ranking [39] for imbalanced settings, termed balanced variable importance (RF BVI), seeking to simplify the learned models. The RF VI of a feature can be loosely interpreted as its effect on the accuracy of a random forest; to account for imbalance, we defined RF BVI as the arithmetic mean of the per-class VI values (see Section 2.5.2 in Additional file 1 for details). Both KW and RF BVI are non-parametric and stable feature selection methods, that is, robust to minor perturbations in the data. Furthermore, in small-sample class-imbalance settings, univariate feature selection, such as with the KW test, can improve predictive performance more than over- and under-sampling [63].

**Fig. 4:**
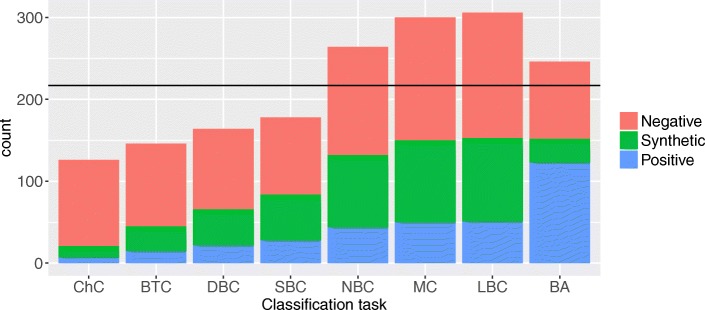
Effects of under- and over-sampling the full dataset with the chosen rates. Each bar represents a one-versus-all classification task (e.g., the leftmost bar is for ChC versus rest). ‘Positive‘ denotes the examples of the class of interest (e.g., ChC in the leftmost bar), ‘Synthetic‘ are the artificial SMOTE examples of the positive class (i.e., the class of interest), while ‘Negative‘ are the kept examples of all remaining classes. The horizontal line shows the size of the original data set (217 examples). For ChC (leftmost bar), for example, applying our sampling method to the full data set containing seven ChC cells (red segment of the bar), would retain 105 (blue segment) out of 210 non-ChC cells and add 14 synthetic ChC cells (green segment), yielding a data set of size 126 (hence the bar is lower than the horizontal line at 217). Except for BA, in all cases the class of interest was the minority class. For BA we performed no undersampling

Most of the classifiers used, as well as the sampling and feature selection methods, require us to specify parameters, such as the number of neighbors for the kNN classifier or the number of majority class instances to remove in undersampling. While learning these from data may improve performance, we opted to avoid additional learning complexity (i.e., increasing the probability of over-fitting) and instead pre-specified all parameters, using mostly the default values from the implementations of the corresponding methods (see Tables 1 and 2) rather than fine-tuning them. For kNN and CART we chose five neighbors (*k*=5) and five instances ($|\mathcal {D}^{l}| = 5$) at leaf nodes, respectively, as we expected lower values to yield overly complex models. For RF BVI we used 20000 trees (*T*=20000) to get stable rankings, while the ranking cut-point value of 0.01 (*bvi*>0.01) for was arbitrary. For over- and under-sampling we devised a heuristic (see Additional file 1: Section 2) to determine the sampling ratios; Fig. 4 illustrates its effects on the class distributions in the different settings. Note that we used the same parameters in all eight classification settings.

**Table 2:** Parameters for feature selection (KW and RF BVI), sampling (SMOTE) and cross-validation (CV)

Method	R Package	Parameters	Learner parameters
KW	stats [38]	*α*=0.05 adjust = FDR	
RF BVI	randomForest [75]	*bvi*>0.01	$T = 20000, m = \sqrt {n}$
SMOTE	mlr [80]	*k*=5	
CV	mlr [80]	*r*=10	k = 10, for ChC k=7

FDR stands for false discovery rate; *r* is the number of CV repetitions; k the number of folds. Learner parameters are the RF parameters used internally for RF BVI

The full learning sequence was therefore: 1) feature selection; followed by 2) data sampling; and finally 3) classifier induction, with steps 1 and 2 being optional (i.e., we also considered not selecting features and not sampling the training data). We evaluated the classification performance with F-measure^13^ [64], a metric useful for assessing the prediction of the class of interest in imbalanced settings, and estimated it with k-fold cross-validation. We ran all three steps of the learning sequence on the k training data sets alone, i.e., without using the test fold (that is, we selected features and sampled data within the cross-validation loop, not outside of it). Since data sampling is stochastic, and a large sampling ratio can change the training set class distribution, we repeated cross-validation ten times when including sampling within the learning sequence. Finally, we identified potentially atypical MC morphologies as those commonly misclassified by different models [45].

In order to classify an interneuron into any of the seven ‘base’ types (i.e, other than the compound BA type), we combined one-versus-all models by assigning the neuron to the type with the most confident model, that is, the one giving the highest probability to its positive class.

Additional file 1 (Section 2) provides relevant details about the methods used, including literature references, precise definitions, the underlying rationale, descriptions of the sampling procedure and F-measure computation, as well as implementation details.

## Results

We first show that some class labels differed from those provided by the neuroscientists in [14] and illustrate reconstruction issues that require care when choosing and computing morphometrics. We then present the classification results and show that accurate models classified MC cells in accordance with the independent classification by the neuroscientists from [14]. Finally, we provide quantitative descriptions of the types, in terms of only a few morphometrics or parsimonious CART and logistic regression models.

### Validating class labels and morphology reconstructions

**Fig. 5:**
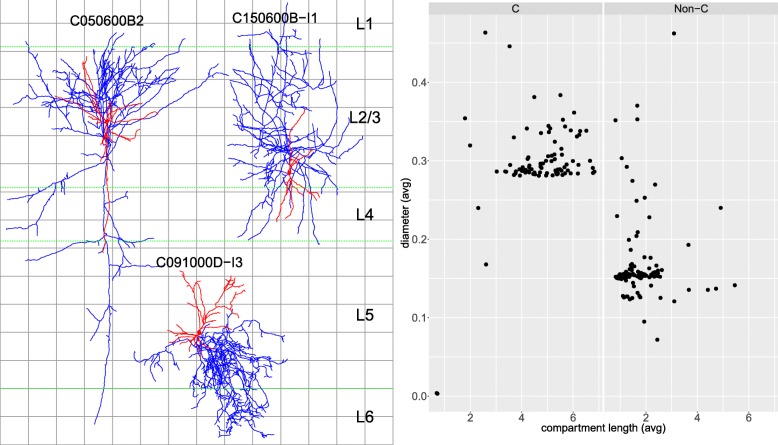
Possible class label and reconstruction issues. Left panel: cells C050600B2 (left), C091000D-I3 (middle), and C150600B-I1 (right) from Table 3, labelled as MC and ChC, respectively, yet only one, three, and one (out of 42) neuroscientists in [14], respectively, coincided with those labels, assigning them instead to the LBC, CB, and CT types. Note that we did not know the location of soma inside their layers; for the MC cells, a soma closer to L1 would mean more extensive axonal arborization in that layer. Axons are drawn in blue with dendrites and somata in red. Dashed green lines indicate layer boundaries from the rat hind-limb somatosensory cortex; L6 is only partially shown. There are 100 *μm* between consecutive grid lines. Right panel: newer reconstructions, whose IDs do not begin with a C, had thinner and shorter segments

For eight out of 20 cells which were also classified by 42 neuroscientists in [14] our class label differed from that given by the majority of the neuroscientists (see Table 3 and Fig. 5, left). There was no strong consensus on the actual type for these cells among the neuroscientists, although cells C050600B2, C091000D-I3, and C170998D-I3 were LBC, CB, and CB, respectively, according to at least 19 of them. For $\frac {5}{19} = 26\%$ of the considered cells no more than five neuroscientists agreed with our class label^14^, suggesting that there might have been many such differing class labels had we been able to compare them for the entire data set.

**Table 3:** Disagreement with our class labels by 42 neuroscientists who participated in [14]

	ID	Layer	Cell type	DF	Agree	**AR**	**CB**	**ChC**	**CR**	**CT**	**HT**	**LBC**	**MC**	**NGC**	**OT**	**UN**
1	blueC040600B2	2/3	MC	CT	0	3	9	0	0	15	2	5	0	0	5	3
2	C050600B2	2/3	MC	LBC	1	0	5	0	0	10	1	20	1	0	2	3
3	C150600B-I1	2/3	MC	CT	1	1	11	0	0	16	0	9	1	0	3	1
4	C091000D-I3	5	ChC	CB	3	3	19	3	0	6	0	6	0	2	2	1
5	C260199A-I3	4	MC	CT	3	0	5	0	0	17	0	6	3	0	4	7
6	C170998D-I3	2/3	NGC	CB	5	1	19	0	0	11	0	0	0	5	4	2
7	C070600B2	4	MC	LBC	11	2	1	0	0	8	0	15	11	0	2	3
8	C090997A-I2	4	MC	CT	12	1	6	0	0	14	0	4	12	0	1	4

Cell type is the label in our data, given according to the classification scheme from [6] while DF (standing for DeFelipe) is the majority label chosen by the neuroscientists, according to the scheme from [14]. Agree is the number of neuroscientists that coincided with our label, while columns to the right show the number of neuroscientists who selected the corresponding DF label (all shown in boldface): AR - arcade; CB - common basket; CR - Cajal-Retzius; CT - common type; HT - horse-tail; OT - other; UN - uncharacterized, meaning that the axonal morphology reconstruction was not sufficient to distinguish the type. The table shows eight out of the 20 interneurons which were classified as ChC, MC, or NGC —the three types common to both classification schemes— in our data yet differently by the majority of neuroscientists (column DF); for the remaining twelve interneurons, the neuroscientists’ majority label matched ours. Cell C040600B2, which was presented to the neuroscientists rotated upside-down, is marked in blue. ID can be used to look the neuron up at Neuromorpho.org

Interestingly, the interneurons could be separated into two groups, one containing cells with their arbors reconstructed at a finer level —with shorter and thinner segments— than those of the other (see Fig. 5, right). We thus avoided using morphometrics sensitive to such fine-grained properties (e.g., the number of segments per branch). However, this difference may have distorted metrics such as tortuosity, since finer reconstructed branches were more tortuous; see Section 3.1 in Additional file 1. 84 cells had at least one multifurcation (a branching point splitting into three or more child branches; at most ten in a single neuron) yet their effect was minimal as we ignored these branching points when computing bifurcation morphometrics, such as mean partition asymmetry or mean bifurcation angle. Two cells seemed to be modified clones of other cells; see Section 3.2 in Additional file 1 for details. We only found two reconstruction anomalies: a 285 *μm* long segment (whereas median length was 2 *μm*), and two axonal arbors that were extremely flat in the Z dimension (less than 80 *μm* deep while median depth was 215 *μm*; ratio of depth to axonal length was below $\frac {1}{100}$ while median ratio was $\frac {1}{62}$). We did not correct these issues nor remove the corresponding neurons.

### Classification

Table 4 shows the best F-measure results for the eight classification settings. The most accurately classified classes were BA, MC, and NBC (shown in green), each with an F-measure ≥0.80, while classifying ChC and BTC cells was difficult (best F-measure 0.50 and 0.44, respectively). The best model for MC performed better than the average neuroscientist in [14] when identifying MC cells, as their average F-measure was 0.72^15^. Accuracy tended to increase with type frequency (F-measure generally increases towards the bottom rows of Table 4), with the exceptions of LBC, which was the third hardest to classify despite being the second most numerous, and BTC, which was the hardest type to classify yet only second least numerous.

**Table 4:** F-measure one-versus-all classification

	Cell Type	Classifier	FSS	Sampling	F-measure	TPR	TNR	Morphom.
1	redChC	RMLR			0.40	2 / 7	209 / 210	11
2		RF		Yes	0.49	2.8 / 7	208.5 / 210	103
3		GBM	KW		**0.50**	3 / 7	208 / 210	15
4		RF	KW	Yes	0.46	3.5 / 7	205.2 / 210	15
5	redBTC	NB			0.35	8 / 15	179 / 202	103
6		GBM		Yes	0.36	5.8 / 15	191.2 / 202	23
7		LDA	KW		**0.44**	6 / 15	196 / 202	7
8		LDA	KW	Yes	0.40	8.8 / 15	181.8 / 202	7
9	orangeDBC	RMLR			0.70	15 / 22	189 / 195	17
10		RF		Yes	0.70	14.6 / 22	189.8 / 195	103
11		RF	RF BVI		**0.72**	13 / 22	194 / 195	6
12		RF	KW	Yes	0.70	15.4 / 22	188.2 / 195	61
13	orangeSBC	CART			0.63	16 / 28	182 / 189	5
14		RF		Yes	0.66	20.6 / 28	174.8 / 189	103
15		NNET	RF BVI		**0.74**	21 / 28	181 / 189	7
16		RF	RF BVI	Yes	0.69	22.5 / 28	173.8 / 189	7
17	greenNBC	CART			0.73	32 / 44	161 / 173	4
18		RF		Yes	**0.81**	36.2 / 44	164 / 173	103
19		GBM	RF BVI		0.78	34 / 44	164 / 173	9
20		GBM	RF BVI	Yes	0.77	37.4 / 44	157 / 173	9
21	greenMC	SVM			0.77	37 / 50	158 / 167	103
22		RF		Yes	0.81	40.2 / 50	158.4 / 167	103
23		RMLR	KW		0.80	38 / 50	160 / 167	22
24		RF	KW	Yes	**0.82**	40.9 / 50	157.8 / 167	62
25	orangeLBC	GBM			0.61	26 / 51	158 / 166	103
26		RF		Yes	**0.67**	29.8 / 51	157.4 / 166	103
27		GBM	RF BVI		0.66	31 / 51	154 / 166	4
28		GBM	RF BVI	Yes	**0.67**	37.4 / 51	142.2 / 166	4
29	greenBA	RF			0.86	106 / 123	76 / 94	103
30		SVM		Yes	0.86	101.9 / 123	80.8 / 94	103
31		SVM	KW		**0.88**	105 / 123	84 / 94	68
32		SVM	KW	Yes	**0.88**	104.2 / 123	84.2 / 94	68

The table shows, for each type, the best F-measure in all four learning settings: with and without sampling, and with and without feature selection. TPR: true positive rate; TNR: true negative rate; the minority class is always the positive one, except for BA; Morphom.: the number of morphometrics in the model. Types are sorted from least to most frequent (e.g., ChC, with only seven examples, is shown uppermost). The best F-measure for each type is typeset in bold. Types with their best F-measure ≥0.75 are shown in green; those with an F-measure ≥0.60 in orange; and the rest in red

Sampling improved the performance of most classifiers, although the largest increase in best F-measure was only 0.03, for the NBC type (see Table 4, row 18). Feature selection increased the best F-measure for BA, DBC, MC, and especially for BTC and SBC (Table 4, rows 7 and 15). RW BVI selected much smaller sets of morphometrics (e.g., 7 for SBC; Table 4, row 15) than KW (up to 68, for BA; Table 4, rows 31-32), allowing, for example, to accurately classify NBC cells using just 9 morphometrics (Table 4, row 19). Further feature pruning by the CART and RMLR models after KW produced parsimonious and accurate models, such as the RMLR model for MC (with an F-measure of 0.80 and 22 morphometrics; Table 4, row 23). See Additional file 1 (Figure S3 to Figure S10) for detailed per-type graphs of classification performance, broken down by classification, feature selection and sampling method.

We achieved best multi-class classification when combining one-versus-all RF models learned after KW feature selection and sampling, with an accuracy of 0.74 (see Figure S11 in Additional file 1 for all accuracies). This produced a notably higher per-class F-measure for LBC (0.75 versus 0.67 in Table 4), lower per-class F-measure for ChC and SBC (0.22 and 0.67 versus 0.50 and 0.74 in Table 4, respectively), and similar values for the remaining types (see Table S9 in the Additional file 1 for the multi-class confusion matrix).

### Validating the MC models

We validated the two most accurate models for MC —RF with sampling and RMLR, both preceded by KW feature selection (see Table 4, rows 22–24)—, by comparing their output to the classification by the neuroscientists from [14], which was not used to train the models.

As Table 5 shows, the models largely agreed with the neuroscientists in [14]. Cells that were considered MC by 13 or less neuroscientists (upper part of Table 5) were also rarely classified as MC by our models, with cells C050600B2, C260199A-I3, and C230998C-I4 never labelled as MC by either model. Both models disagreed with the neuroscientists on cells C040600B2 and C090997A-I2 —the former was, however, shown to the neuroscientists rotated upside-down, which may account for so few votes for MC— and RF disagreed on cell C150600B-I1, considering it MC 22 out of 30 times. On the other hand, cells that were MC according to 14 or more neuroscientists (lower part of Table 5) were always classified as MC by the models, except for C061000A3, which RMLR never classified as MC.

**Table 5:** Classification of MC cells by the neuroscientists in [14] and our two most accurate models, RF and RMLR

	ID	Layer	RF	RMLR	MC	Non-MC	UN
1	blueC040600B2	L2/3	29	23	0	39	3
2	redC050600B2	L2/3	0	0	1	38	3
3	C150600B-I1	L2/3	22	1	1	40	1
4	redC260199A-I3	L4	0	0	3	32	7
5	C070600B2	L4	12	0	11	28	3
6	C090997A-I2	L4	30	19	12	26	4
7	redC230998C-I4	L4	0	0	13	25	4
8	C190997A-I1	L4	30	30	14	26	2
9	C290500B-I3	L2/3	30	30	18	20	4
10	C150501A-I3	L5	30	30	22	6	14
11	C060400C1	L2/3	30	30	24	18	0
12	C290500C-I4	L5	30	30	26	15	1
13	C061000A3	L4	30	0	34	6	2
14	C100501A3	L2/3	30	30	36	3	3
15	C050896A-I	L5	30	30	37	4	1
16	C070301B2	L6	30	30	37	4	1
17	C180298B-I3	L5	30	30	38	3	1

MC is the number of neuroscientists who classified the cell as MC, Non-MC the number of those who assigned it to another type, and UN the number of those who considered that the axonal morphology reconstruction was not sufficient to distinguish the type. RF and RMLR show the number of times (out of 30) that RF and RMLR classified the cell as MC. Cells that were never classified as MC by both models are marked in red. Cell C040600B2, which was presented to the neuroscientists rotated upside-down, is marked in blue. ID can be used to look the neuron up at Neuromorpho.org

**Fig. 6:**
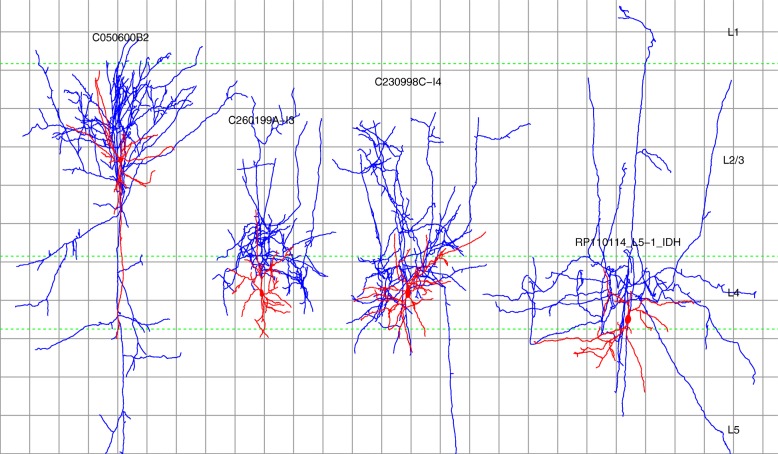
MC cells that were classified as non-MC by the two most accurate models. Cells C050600B2, C260199A-I3, and C230998C-I4 were classified as MC by only one, three, and 13 neuroscientists in [14], respectively. Cells C260199A-I3 and C230998C-I4 do not reach L1 unless their actual soma was located near the top of L4, although tissue shrinkage may have reduced their height by around 10%. Axons are drawn in blue with dendrites and somata in red. Dashed green lines indicate layer boundaries from the rat hind-limb somatosensory cortex. There are 100 *μm* between consecutive grid lines

Figure 6 shows the four cells that were considered MC at most six (out of 30) times by both RF and RMLR. These include the cells C050600B2, C260199A-I3, C230998C-I4 (shown in red in Table 5), classified as MC by only one, three, and 13 neuroscientists, respectively. These cells may correspond to atypical MC morphologies.

### Feature selection

For all types except for ChC and BTC, we achieved at least moderately accurate (F-measure ≥0.65) models using few morphometrics (see Table S5 in the Additional file 1). Below we describe the BA, NBC, DBC, SBC, and SBC types in terms of the morphometrics selected with RF BVI, and the MC type in terms of those selected with KW followed by CART and RMLR embedded feature selection (this yielded more accurate models for MC than RF BVI). We also describe the BA and MC types in terms of accurate (F-measure ≥0.75) and parsimonious CART and logistic regression (RMLR) models. Finally, we complement each type description with some of the best-ranked morphometrics according to the KW test, and conclude with a summary of feature selection. We begin with the most accurately classified type, BA, and proceed towards the least well discerned ones, ChC and BTC. See Additional file 1 for the full list of KW- and RF BVI-selected morphometrics (Tables S7 and S8, respectively), along with the corresponding *p*-values and RF BVI values.

#### BA characteristics

**Fig. 7:**
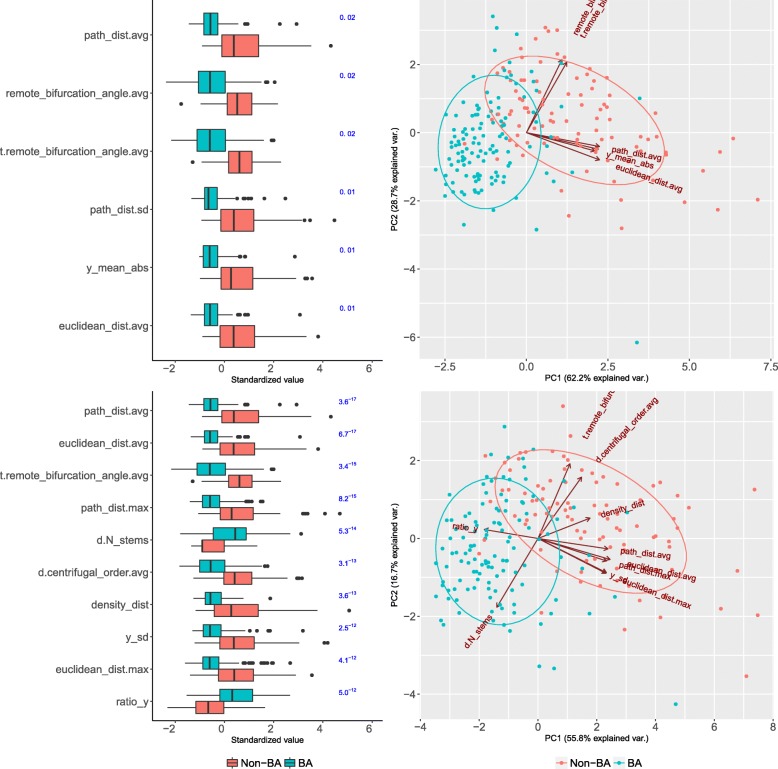
Relevant morphometrics for the BA type. Top left: per-type boxplots for the six morphometrics selected with RF BVI (RF BVI values shown, in blue, to the right). The most relevant morphometrics, mean arborization distance to soma (path_dist.avg), and mean remote bifurcation angle (remote_bifurcation_angle.avg), are shown in the upper part of the panel. Top right: a biplot of these six morphometrics, with the data projected onto the two principal components, found with principal component analysis (vectors represent morphometrics and the angles between them are indicative of their pairwise correlation). All morphometrics were correlated with either path_dist.avg or remote_bifurcation_angle.avg. Bottom left: the ten most relevant morphometrics according to KW, after removing those with absolute correlation >0.90 with a better ranked morphometric, with the KW *p*-values shown, in blue, to the right of the boxplot. These morphometrics included those relative to arborization distance from soma (e.g., euclidean_dist.avg, path_dist.avg), remote bifurcation angles (t.remote_bifurcation_angle.avg), the number of dendritic trees (d.N_stems), and axonal arborization along the radial direction (ratio_y). In addition to having sharper bifurcation angles and arborizing closer to the soma, especially in the radial direction, BA cells had more dendritic trees than non-BA cells

**Fig. 8:**
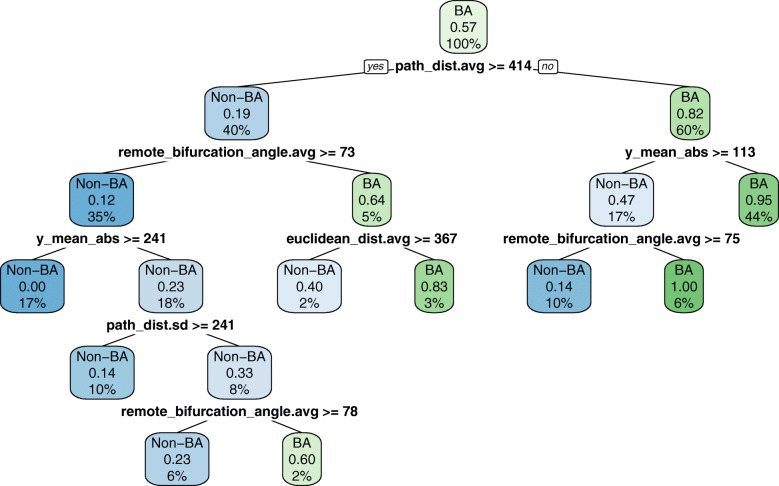
CART model (F-measure value of 0.85) for BA derived from the six morphometrics selected with RF BVI. Most of the BA cells (i.e., those contained in the two rightmost tree leaves) have a path_dist.avg <414 and either y_mean_abs <133 or remote_bifurcation_angle.avg <75°, meaning that they arborize close to the soma, especially vertically, whereas if they do arborize further vertically (as some LBC cells do), they have sharper bifurcation angles. Each box represents a split in the data set, indicating: (a) its majority type (BA is the majority type overall and hence it is shown in the root node of the tree (i.e., the initial split)); (b) proportion of positive examples (BA cells represent 57% of the data set and hence 0.57 in the root node; they present 95% of the samples in the rightmost node); and (c) the percentage of the data set reaching the split (100% of the data passes through the root split; 44% of the data set reaches the rightmost node)

Six axonal morphometrics selected by RF BVI (Fig. 7) sufficed to accurately (with an F-measure of 0.86) distinguish BA cells. These morphometrics captured two properties only: remote branching angle and arborization distance from soma. Indeed, BA cells had sharper remote bifurcation angles and arborized closer to the soma, especially in terms of vertical distance (Fig. 7). While LBC cells can extend vertically far from the soma ([6, 16]; their average height in our sample was 1020*μm* ±327*μm*, versus 603 *μm*± 190 *μm* for the NBC and SBC together), it seems that most of their arbor is nonetheless located near the soma, with radially distant ramifications being rather sparse. The CART and RMLR models derived from the six RF BVI-selected morphometrics were accurate (F-measure of 0.85 and 0.83, respectively) and interpretable (e.g., [19] used CART to relate mRNA expression to neuro-anatomical type). The CART model, for example, is a set of rules such as “all cells with path_dist.avg < 414 and y_mean_abs < 133 are BA cells”. The models are presented in Fig. 8 and Table 6.

**Table 6:** Logistic regression (F-measure of 0.83) model for BA derived from the six morphometrics selected with RF BVI, with the ***β*** estimated from the standardized data set, and BA being the positive class

Morphometric	*β*
remote_bifurcation_angle.avg	−2.1×10^−1^
euclidean_dist.avg	−1.2×10^−2^
y_mean_abs	−3.3×10^−3^
path_dist.sd	−1.5×10^−3^
path_dist.avg	−2.0×10^−4^

Interpretation is straightforward; for example, according to the model, a 7.33°increase in the average bifurcation angle of a cell reduce the log-odds of BA by 0.21

The KW test identified a further 63 morphometrics, including 26 dendritic ones, that differed between the BA and non-BA cells, yet using them barely improved the F-measure achieved with the six RF BVI-selected morphometrics alone (from 0.86 to 0.88). Interestingly, the number of dendritic trees was among the most relevant morphometrics, with BA cells having more dendritic trees than non-BA ones (Fig. 7). Although some basket cells have curved axon terminals [9], t.tortuosity.avg was only 47-th most relevant morphometric according to KW, suggesting that we may need a more appropriate morphometric to capture the curved property of basket terminal branches. Axonal properties that did not differ for BA cells included average branch length, arbor length and initial direction (whether towards pia or the white matter).

#### MC characteristics

**Fig. 9:**
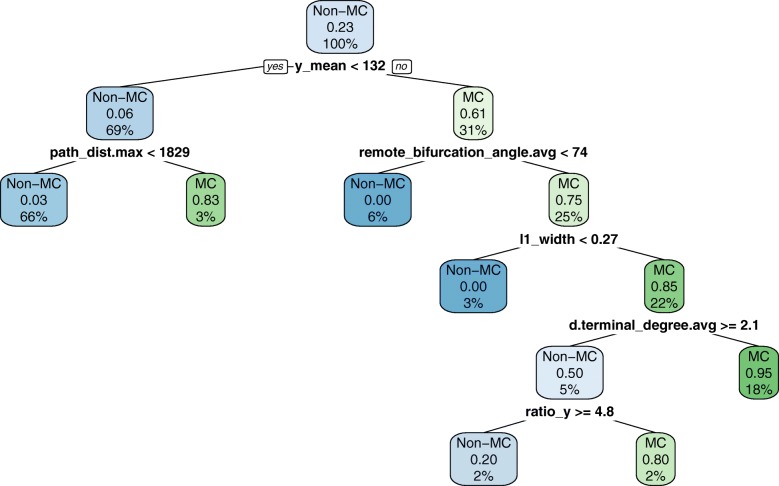
CART model for MC, with an F-measure value of 0.75. Most MC cells (rightmost leaf) have a y_mean ≥132 (their axons mainly arborize above the soma), remote_bifurcation_angle.avg ≥ 74°, l1_width ≥0.27 and dendritic terminal degree <2.1. Each box represents a split in the data set, indicating: (a) its majority type (Non-MC is the majority type overall and hence it is shown in the root node of the tree (i.e., the initial split), whereas MC is the majority type in the rightmost split); (b) the proportion of positive examples (MC cells represent 23% of the whole data set and hence 0.23 in the root node; they present 95% of the samples in the rightmost node); and (c) the percentage of the data set reaching the split (100% of the data passes through the root split; 18% of the data set reaches the rightmost node)

**Fig. 10:**
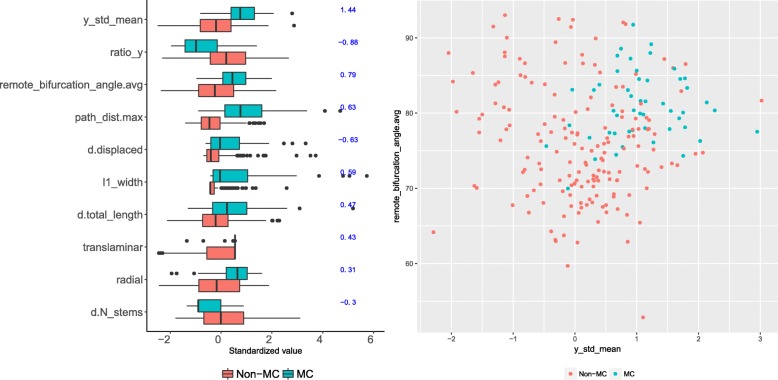
Relevant morphometrics for the MC type. Left: ten morphometrics with strongest ***β*** in the KW + RMLR model (***β*** shown, in blue, to the right of the boxplot; full model in Additional file 1, Table 6). Largely positive y_std_mean (top of the boxplot) indicates that MC cells preferentially arborized above the soma. Having longer dendritic arbors (d.total_length) but less dendrites (d.N_stems) means that MC cells had longer individual dendritic trees; these arbors were displaced from the axonal ones (d.displaced), which were often radially oriented (radial). Right: MC cells mainly arborize above the soma (y_std_mean) and have wide bifurcation angles (remote_bifurcation_angle.avg)

The six morphometrics selected by CART (following KW selection) allowed for classifying MC cells with an F-measure of 0.75. According to this model, a typical MC cell’s axon arborized far above the soma (y_mean), widely in layer L1, and bifurcated in wide angles. The model is described in Fig. 9. Using 22 morphometrics, including seven dendritic ones, KW + RMLR was more accurate (F-measure of 0.80) and uncovered additional MC properties, such as longer dendritic trees, displaced from axonal arbors, which in turn were moderately radial (see Fig. 10). This agrees with [6] and [57], who reported elaborate dendrites, 1013 ±503 *μm* axonal width in L1, and average tilt angles of 80 degrees. It also contrasts with the above description of BA cells, which arborized vertically close to the soma, had shorter bifurcation angles, and many dendritic trees. This is illustrated in Fig. 10, which plots MA, BA and all other types using the two most useful morphometrics for BA.

KW selected 40 additional morphometrics, including 17 dendritic ones, with the strongest difference for path_dist.avg and y_mean (see Table S7 in Additional file 1). MC cells often had bitufted dendrites (also reported by [6]) and axons originating above the soma.

#### NBC characteristics

**Fig. 11:**
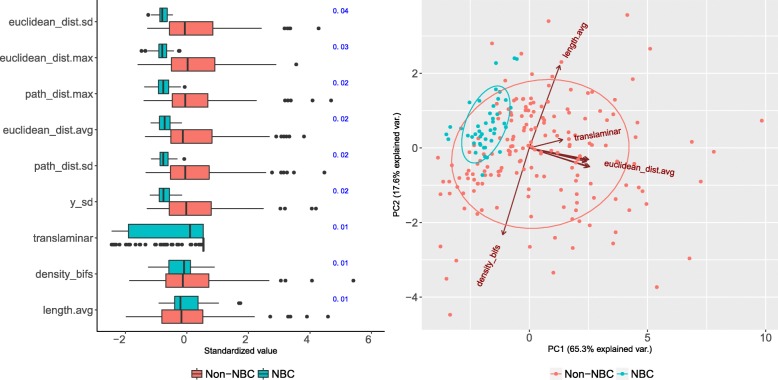
Relevant morphometrics for the NBC type. Left: per-type boxplots for the nine morphometrics selected with RF BVI (RF BVI values shown, in blue, to the right). For most NBC cells, the axon never arborized far from the soma (low euclidean_dist.max; top part of the panel) nor outside of its cortical layer (low translaminar). Although selected by RF BVI, length.avg and density_bifs, the box-plots (bottom part) show that these morphometrics were not univariately useful. Right: the nine selected morphometrics separate the NBC cells from non-NBC ones. The biplot shows the data projected onto the two principal components, found with principal component analysis, with the vectors representing the morphometrics and the angles between them indicative of their pairwise correlation. Besides branch length (length.avg), translaminar reach (translaminar), and arborization density (density_bifs), all selected morphometrics are related to arborization distance from soma. They correspond to the vectors pointing towards the right; only euclidean_dist.avg is annotated to avoid overlapping

Nine axonal morphometrics selected by RF BVI allowed an accurate (F-measure 0.78) classification of NBC cells (see Fig. 11). Six of these morphometrics were related to arborization distance from soma; the rest to translaminar reach, branch length, and arbor density.

KW identified a larger and more diverse set of 48 morphometrics, including 21 dendritic ones, that differed for NBC cells (see Table S6 in Additional file 1), yet using all of them slightly decreased performance with respect to using only the nine RF BVI-selected morphometrics (F-Measure from 0.78 down to 0.75). In addition to arborization distance from soma and translaminar reach, relevant morphometrics included axonal terminal degree, arbor eccentricity, partition asymmetry, terminal branch length, and whether the dendrites were bitufted.

#### DBC, SBC and LBC characteristics

**Fig. 12:**
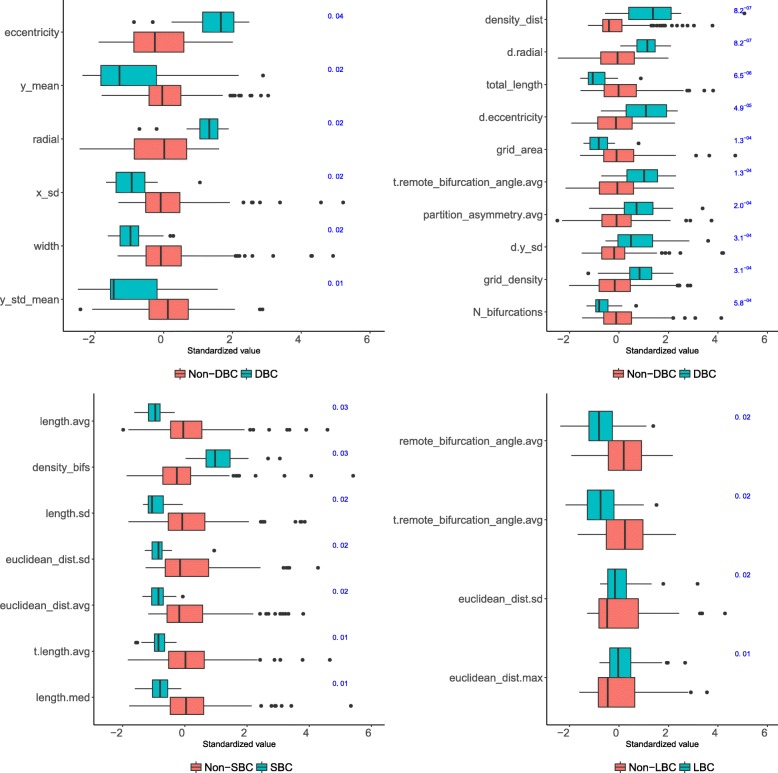
Relevant morphometrics for the DBC (above) and SBC and LBC (below) types. Top left: per-type boxplots for the morphometrics selected with RF BVI (RF BVI values shown, in blue, to the right). The axonal arbor of a typical DBC cell was radially oriented (high radial and eccentricity values), rather than circular, it did not spread far tangentially (low x_sd and width), and was mainly located below the soma (low y_std_mean and y_mean). Top right: the ten most relevant morphometrics according to KW, after removing those already shown in the left panel and those with an absolute correlation >0.90 with a better ranked morphometric (KW *p*-values shown, in blue, to the right). DBC cells’s dendrites were bipolar/bitufted (d.insert.radial, not shown), arborized along the radial axis (d.radial) and reached far radially (d.y_sd), while their axonal arbors were short (total_length), with wide terminal bifurcation angles (t.remote_bifurcation_angle.avg). Bottom left: per-type boxplots for the morphometrics selected with RF BVI for SBC (RF BVI values shown, in blue, to the right). SBC cells had short branches (low length.avg) and dense, local arbors (low density_bifs and euclidean_dist.avg). Bottom right: per-type boxplots for the morphometrics selected with RF BVI for LBC (RF BVI values shown, in blue, to the right). LBC cells had sharp bifurcation angles

DBC cells were classified with moderate accuracy (F-measure 0.72) with the five morphometrics selected by RF BVI, all related to axonal arbor eccentricity, distribution along the Y axis, and width (see Fig. 12). While KW identified 61 significantly different morphometrics for DBC —more than for SBC, NBC, and LBC, even though these were more numerous than DBC— using all of those morphometrics did not improve DBC classification (F-measure dropped to 0.70). The most relevant ones were related to the radial arborization of both the axon and the dendrites (Fig. 12). Interestingly, KW selected more (26) dendritic morphometrics for DBC than for any other type.

For SBC we achieved an 0.73 F-measure with the seven RF BVI-selected morphometrics, related to mean branch length, arbor density, and arborization distance from soma (see Fig. 12). KW selected 39 morphometrics, although using them did not improve with respect to using RF BVI-selected ones alone (F-measure from 0.73 down to 0.67). Relevant morphometrics included y_sd, related to radial arborization extent, and the maximal arborization distance from the soma (euclidean_dist.max).

LBC cells were classified with an F-measure of 0.66 with the four morphometrics selected with RF BVI, related only to remote bifurcation angles and arborization distance from soma (see Fig. 12). According to KW, the remote bifurcation angle was the most significant morphometric, with a *p*-value of 3.7×10^−8^, followed by remote tilt angle, median terminal branch length, grid_area and the number of dendrites (see Table S7 in Additional file 1). KW identified only 32 relevant morphometrics for LBC, much less that for other numerous types; using all these morphometrics reduced the best F-measure to 0.62.

#### BTC and ChC characteristics

For BTC, only seven morphometrics were relevant according to KW, with dendritic polarity and the standard deviation of branch length (length.sd), among the most significant ones. For ChC, the relevant properties according to KW included arbor density (density_bifs, grid_mean), mean branch length, the number of short vertical branches, and terminal degree.

#### Summary

KW identified more relevant morphometrics for the more numerous types, with the exceptions of LBC (second most numerous, yet only sixth most features) and DBC (sixth most numerous, yet third most features). Dendritic morphometrics represented 30–40% of the relevant ones, except for ChC (a single dendritic morphometric out of seven relevant ones; see Table S7 in Additional file 1). 11 dendritic and four axonal morphometrics were not relevant for any type, and are possibly useless for interneuron classification: dendritic bifurcation angles, tortuosity, and radial and tangential arbor distribution, and axonal torque angle and tangential arbor distribution. Dendritic tree length and d.displaced, however, were relevant for six out of eight types. Custom-implemented morphometrics represented between 47% and 72% of the selected morphometrics. Only two custom-implemented morphometrics (ratio_x and x_mean_abs) were not useful for any type, while translaminar and y_sd were relevant for six types.

## Discussion

We obtained accurate models for the NBC, MC, and BA types and moderately accurate ones for DBC, SBC, and LBC. The best MC model was better than the average neuroscientist in [14] and was outperformed by only three out of 42 of them (see Section 6 in Additional file 1). The best BA model was even more accurate, correctly identifying 105 out of 123 BA cells (see Table 4). These models, along with the model for NBC, would probably be useful for the definitive automatic classifier envisioned by [14] to replace neuroscientists in this task. The remaining models were probably not good enough: the next best model correctly identified only 20 out of 28 SBC cells (see Table 4). The main limiting factor seems to have been sample size: with the exception of LBC, more numerous types were classified more accurately; indeed, we only had 28 SBC, 22 DBC, 15 BTC and seven ChC cells. Taking sample sizes into account, moderate F-measure values suggest that the DBC and SBC types are morphologically distinct and we expect that around 50 cells (a count close to that of NBC and MC cells) would suffice to accurately classify them. The LBC type was relatively hard to classify. Either we have missed to quantify its distinctive morphometrics —there were less relevant morphometrics for LBC than for other numerous types— or its morphology is not sufficiently distinct when contrasted to the other types merged together. Distinguishing across layers (e.g., L2/3 LBC, L4 LBC, etc.) might decompose it into morphologically distinct subtypes.

One explanation for the differences between our class labels and the classification from [14] shown in Table 3 is that ours were ultimately determined by the presence of spiny boutons and dendritic spines (MC), short vertical rows of boutons (ChC), or a high density of small boutons (NGC). Indeed, for [57] spiny boutons, along with axonal spread in L1, are an essential (mandatory) characteristic of MC cells. Yet, ChC, MC and, to a lesser degree, NGC morphologies are often identifiable by axonal and dendritic geometry alone [14] suggesting that their arborization patterns are distinct. Thus, while cells in Table 3 might be meeting fine-grained criteria for MC, ChC, and NGC membership, their high-level morphologies are atypical, as most of the 42 neuroscientists considered that they did not belong to those types. It is hard for a model to correctly classify such cells, unless some morphometrics are correlated with the fine-grained features. Thus, there might be a limit to how well the classification by [6] could be replicated by a model trained on morphological reconstructions. However, even when the MC models failed to recover the class label, their output may have been sensible, as it was often consistent with the classification by the 42 neuroscientists (see Table 3). MC cells classified as not MC by accurate models might thus correspond to atypical MC morphologies.

An alternative, but less likely, explanation for the difference is that some class labels had been wrongly assigned, without following the pre-specified criteria. In that case, wrong labels would have biased the models as well as their performance estimates [65]. Instead of assuming that all class labels are correct, as we did, they can be estimated together with classifier learning (Frénay and Verleysen, 2014), although this makes the learning problem more difficult.

Additional morphometrics might further improve the results. We consider that quantifying Petilla features related to arborization patterns would be useful, especially for scarce types such as ChC. Some of our custom-implemented morphometrics may have been too simple (e.g., only branches extending no more than 50 *μm* vertically were considered short and vertical) to adequately capture the complexity of these features, and could be elaborated. Type-specific morphometrics, such as the extent of axonal arborization in layer L1 for MC cells, incorporated prior knowledge about the types into the models. Note that such underlying knowledge may be disputed: e.g., [14] do not require an MC cell to reach layer L1, while [57] consider it an essential, mandatory feature, as do [13], except for L6 MC cells. It would be interesting to study the robustness of standard morphometrics to reconstruction issues such as inconsistent branch granularity and then develop robust alternatives. For example, t.tortuosity.avg might have better captured the ‘curved terminal branches’ feature of the BA type had some cells’ branches not been reconstructed in finer detail than those of others, thus increasing their tortuosity (see Section 3.1 in Additional file 1). While at least 21 analyses available in L-Measure would have not been robust to reconstruction granularity inconsistency in this data set, they are nonetheless used for neuron classification (e.g., [66],). Thus, a software tool that implements robust morphometrics could be useful for practitioners.

The small feature subsets and parsimonious models that allowed (moderately) accurate classification serve as summaries of the types’ morphological characteristics. Most types can be summarized in terms of simple morphometrics, related to arborization distribution with respect to the soma (e.g., path_dist.avg), its vertical direction (e.g., y_std_mean), branching angles (remote_bifurcation_angle.avg), or the number of dendrites (d.N_stems), and a few elaborate ones, such as the arborization extent in L1 (l1_width).

We have presented eight separate type-specific models and combined them to classify a given interneuron by choosing the type with the most confident one-versus-all model. An alternative is to learn a hierarchy of classifiers by grouping types into ‘super types’ such as BA: one would first classify a cell as BA or non-BA and then, if classified as BA, distinguish among LBC, NBC, and SBC types, and among the remaining types otherwise. Rather than learning the hierarchy from data, one might predefine it; useful ‘super-types’ could be formed, for example, by grouping according to axonal target area — a dendrite-targeting type would be composed of BP, BTC, DBC and NGC cells [6].

Note that we have learned the models from juvenile rat somatosensory cortex interneurons and these models might be less effective if applied to classify other species’ or brain area cells, especially because metric variables, such as those related to distances from the soma and arbor size, are affected by these factors. Doing so would also require appropriate laminar thickness metadata in order to quantify laminar extent. The presented supervised classification approach could easily be extended to allow the discovery of new types: since models such as logistic regression can quantify the confidence in their prediction, one could consider discovering types by clustering [67] cells that the model cannot reliably assign to any of the a priori known types.

## Conclusion

We used 217 high-quality morphology reconstructions of rat interneurons to learn models for eight interneuron types. We have proposed and implemented morphometrics that quantify relevant interneuron properties such as laminar distribution and arbor extent in L1, dendritic polarity, arbor orientation, and whether or not the dendrites are displaced from the axon. We carefully selected standard metric and topological morphometrics, omitting those that are not robust to reconstruction granularity. We applied well-known classification algorithms and learned accurate (F-measure values above 0.80), competitive with neuroscientists, models for the BA, MC, and NBC types, and moderately accurate (F-measure above 0.70) models for the DBC and SBC types, although we had less than 30 cells of the latter two types. We characterized the types in terms of parsimonious CART (for BA and MC) and logistic regression (for BA) models that can be interpreted by neuroscientists, and in terms of small sets of relevant morphometrics: no more than nine morphometrics sufficed for an at least moderately accurate classification of the DBC, SBC, NBC, MC and BA types. The most relevant morphometrics were related to axonal arborization distance from the soma and bifurcation angles while most dendritic morphometrics were not relevant. Differences between our class labels and those by 42 leading neuroscientists from [14] suggest that it might be difficult to perfectly replicate the classification by [6] without access to fine-grained morphological features. However, even when failing to recover the original label, the models’ output seemed sensible as it often matched the classification by 42 leading neuroscientists. We computed all the morphometrics with open-source software and our code and data are publicly available. This study showed that with quality reconstructions, a careful selection of morphometrics and an informed machine learning approach, accurate models can be learned from relatively few examples. We speculate that 50 cells could suffice for learning accurate models for the DBC and SBC types. This study also illustrated minor reconstruction issues present in a curated set of high-quality morphologies.

Achieving accurate automatic classification for all established morphological types will require more labeled interneurons to train the models with, especially for scarce types such as ChC. In the short term, this may require leveraging the reconstructions from Neuromorpho.org. Automated checks of morphology, such as those performed by NeuroSTR (e.g., whether a bifurcation angle is too wide to be plausible), could help filter useful reconstructions, while developing morphometrics robust to different types of variability (e.g., in reconstruction granularity) might facilitate combining diverse data. Aggregating cells labeled in different laboratories could be problematic if these class labels have been assigned following different criteria, and the labels might need to be validated by multiple neuroscientists. Classification criteria that give importance to fine-grained morphological features, such as bouton distribution, would imply a limit to attainable classification accuracy, unless we can discover morphometric correlates of such features. Finally, morphometrics that quantify complex arborization patterns could be especially useful for the less numerous types. In the long run, we expect efforts by the Human Brain Project, the Allen Institute for Brain Research, and NeuroMorpho.Org to provide many high-quality morphologies. Given such data, we consider that the methodology presented in this article can provide an accurate automatic classification into established morphological types.

## Additional file

Additional file 1 Towards a supervised classification of neocortical interneuron morphologies. (PDF 1906 kb)

## Abbreviations

ADA: AdaBoost
AR: Arcade
BA: Basket
BTC: Bitufted
CART: Classification and regression trees
CB: Common basket
ChC: Chandelier
CR: Cajal-Retzius
CT: Common type
CV: Cross-validation
DBC: Double bouquet
HT: Horse-tail
kNN: *k*: nearest neighbors
KW: Kruskal-Wallis
LBC: Large basket
LDA: Linear discriminant analysis
MC: Martinotti
NB: Gaussian naïve Bayes
NBC: Nest basket
NNET: Single-layer neural network
OT: Other
RBF: Radial basis function
RF: Random forest
RF BVI: random forest balanced variable importance
RMLR: Lasso regularized logistic regression
SBC: Small basket
SMOTE: Synthetic minority over-sampling technique
SVM: Support vector machine
UN: Uncharacterized

## Footnotes

1
http://celltypes.brain-map.org/

2 While [6] describe nine interneuron types in L2/3 to L6, we lacked enough bipolar and neurogliaform cells to learn classifiers for them. We also grouped small, nest, and large basket cells into a separate, basket type.

3 NeuroSTR is an open source library developed in our research group in the context of the Human Brain Project [68]. Its online repository is at https://github.com/ComputationalIntelligenceGroup/neurostr.

4 We restricted this analysis to the MC type as only for MC we could compare it to an independent classification by neuroscientists in [14].

5 Online repository at https://github.com/ComputationalIntelligenceGroup/bbp-interneurons-classify.

6 We used Table 1 in [13] to map between the two schemes. While the LBC was also common to the two schemes, Table 1 in [13] maps it to the common basket type in [14].

7 The online repository: https://github.com/BlueBrain/NeuroM.

8 [13] used 1009 digitally reconstructed cells; the 228 cells that we use are the interneurons that they classified on the basis of morphological parameters, as shown in Additional file 1: Figure S2 of that paper.

9 One of these 20 cells, C040600B2, was shown to the neuroscientists rotated upside-down, which may have affected how they classified it.

10 We found that in the study by [14], the shortest axon which allowed at least half of the 42 neuroscientists involved to characterize an interneuron (i.e., to consider that the neuron can be classified) was 2805 *μ**m*, with the next shortest being 3197 *μ**m*.

11 We considered all 228 cells when contrasting our class labels to those from [14].

12 In our binary classification settings the Kruskal-Wallis test corresponds to its special case for two samples, the Wilcoxon–Mann–Whitney test [69, 70]. We keep the term Kruskal-Wallis as that is the implementation that we used (R function kruskal.test).

13 The F-measure is the harmonic mean of precision and recall of a single class. In the ChC versus non-ChC setting, for example, these correspond to the percentage of cells classified as ChC which truly are ChC (precision), and the percentage of ChC cells correctly identified as ChC (recall). See Section 2.8 in Additional file 1 for details.

14 We are ignoring cell C040600B2, which was shown to the neuroscientists rotated upside-down (this may have affected how they classified it), hence five out of 19 and not six out of 20.

15 This value was not reported in [14]; instead we computed it from data from that study, taking into account only cells that could be clearly classified into a type. See Section 6 in Additional file 1 for details.
