# Supplementary material for: Towards a supervised classification of neocortical interneuron morphologies
Source: BMC Bioinformatics. 2018 Dec 17;19:511. doi: 10.1186/s12859-018-2470-1 (PMC6296106; doi:10.1186/s12859-018-2470-1)
Supplement: Supplementary file 1 — Towards a supervised classification of neocortical interneuron morphologies. (PDF 1906 kb) [file 12859_2018_2470_MOESM1_ESM.pdf]

Towards a supervised classification of neocortical interneuron morphologies

Supplementary material

Bojan Mihaljević[1], Pedro Larrañaga[1], Ruth Benavides-Piccione[2], Sean Hill[3], Javier DeFelipe[2], and Concha Bielza[1]

[1]Computational Intelligence Group, Departamento de Inteligencia Artificial, Universidad Politécnica de Madrid, Boadilla del Monte, 28660, Spain
[2]Laboratorio Cajal de Circuitos Corticales, Universidad Politécnica de Madrid and Instituto Cajal (CSIC), Pozuelo de Alarcón, 28223, Spain
[3]Laboratory for the Neural Basis of Brain States, Krembil Centre for Neuroinformatics, Toronto, Canada and Blue Brain Project, École Polytechnique Fédérale de Lausanne, Geneva, Switzerland

September 24, 2018

Contents

1 Morphometrics **2**
- 1.1 NeuroSTR morphometrics . . . 2
- 1.2 Custom-implemented . . . 3
  - 1.2.1 Distribution along X and Y axes . . . 3
  - 1.2.2 Laminar distribution . . . 4
  - 1.2.3 MC arborization pattern . . . 5
  - 1.2.4 ChC arborization pattern . . . 5
  - 1.2.5 Arbor density . . . 5
  - 1.2.6 Dendritic bipolarity . . . 5
  - 1.2.7 Displaced dendritic arbor . . . 6
- 1.3 Implementation . . . 6

2 Supervised classification **6**
- 2.1 Overview . . . 6
- 2.2 Notation and terminology . . . 6
- 2.3 Supervised classifiers . . . 6
  - 2.3.1 CART . . . 6
  - 2.3.2 Random forest . . . 7
  - 2.3.3 AdaBoost . . . 7
  - 2.3.4 Naive Bayes . . . 7
  - 2.3.5 k-nearest neighbors . . . 7
  - 2.3.6 Regularized logistic regression . . . 8
  - 2.3.7 Single-layer neural network . . . 8
  - 2.3.8 Support vector machine . . . 8
  - 2.3.9 Linear discriminant analysis . . . 9
- 2.4 Undersampling and oversampling . . . 9
- 2.5 Feature selection . . . 9
  - 2.5.1 Kruskal-Wallis test . . . 9
  - 2.5.2 RF variable importance . . . 9

2.6 Detecting mislabelled examples 10
2.7 Unsupervised preprocessing 10
2.8 Assessing performance 10
2.9 Parameters 11
2.10 Implementation 11

3 Morphology quality **12**
3.1 Reconstruction differences 12
3.2 Two cloned cells 13

4 Feature selection results **13**

5 Classification results **17**
5.1 Multi-class 17

6 Neuroscientists' F-measure for the MC type **18**

1 Morphometrics

1.1 NeuroSTR morphometrics

We computed 'standard' morphometrics with the NeuroSTR neuroanatomy library (see Table 1). These include branch length and bifurcation angles, arbor height and width, and topological features such as vertex ratio. We mainly summarized part-of-tree analyses (i.e., those computed for a section of an arbor, such as a branch or segment) by computing their average, using the median, standard deviation, or maximum statistics only when we deemed it justified (e.g., for maximum arbor distance to soma). We also computed some morphometrics specific to axonal terminal branches (e.g., mean terminal branch length).

Table 1: NeuroSTR morphometrics. For part-of-tree morphometrics, suffixes avg, med, sd, and max denote the mean, median, standard deviation, and maximum, respectively. Detailed documentation for NeuroSTR features is available online: https://computationalintelligencegroup.github.io/neurostr/doc/measures/prebuilt.html.

Morphometric	Axon	Terminal	Dendrite
`centrifugal_order.avg`	✓		✓
`centrifugal_order.max`	✓		✓
`centrifugal_order.sd`	✓		✓
`euclidean_dist.avg`	✓		✓
`euclidean_dist.max`	✓		✓
`euclidean_dist.sd`	✓		✓
`height`	✓		✓
`length.avg`	✓	✓	✓
`length.med`	✓	✓	✓
`length.sd`	✓		✓
`N_bifurcations`	✓		✓
`N_stems`			✓
`partition_asymmetry.avg`	✓		✓
`path_dist.avg`	✓		✓
`path_dist.max`	✓		✓
`path_dist.sd`	✓		✓
`remote_bifurcation_angle.avg`	✓	✓	✓
`remote_tilt_angle.avg`	✓	✓	✓
`remote_torque_angle.avg`	✓	✓	✓
`terminal_degree.avg`	✓		✓
`tortuosity.avg`	✓	✓	✓
`tortuosity.med`	✓		✓
`total_length`	✓		✓
`tree_length.avg`			✓
`vertex_ratio`	✓		
`width`	✓		✓

1.2 Custom-implemented

We used 48 axonal and dendritic custom-implemented morphometrics (see Table 2).

Table 2: Custom morphometrics.

Type	Morphometric	Axon	Dendrite
Arbor density	`density_area`	✓	✓
Arbor density	`density_bifs`	✓	✓
Arbor density	`density_dist`	✓	✓
ChC arborization pattern	`short_vertical_terminals`	✓	
Dendritic displaced	`displaced`		✓
Dendritic polarity	`insert.eccentricity`		✓
Dendritic polarity	`insert.radial`		✓
Laminar	`l1_prob`	✓	
Laminar	`translaminar`	✓	✓
MC arborization pattern	`l1_bifs`	✓	
MC arborization pattern	`l1_gx`	✓	
MC arborization pattern	`l1_gxa`	✓	
MC arborization pattern	`l1_width`	✓	
XY distribution / Axon origin	`axon_above_below`	✓	
XY distribution / Axon origin	`axon_origin`	✓	
XY distribution / Grid	`grid_area`	✓	✓
XY distribution / Grid	`grid_density`	✓	
XY distribution / Grid	`grid_mean`	✓	✓
XY distribution / Moments	`ratio_x`	✓	✓
XY distribution / Moments	`ratio_y`	✓	✓
XY distribution / Moments	`x_mean`	✓	✓
XY distribution / Moments	`x_mean_abs`	✓	✓
XY distribution / Moments	`x_sd`	✓	✓
XY distribution / Moments	`y_mean`	✓	✓
XY distribution / Moments	`y_mean_abs`	✓	✓
XY distribution / Moments	`y_sd`	✓	✓
XY distribution / Moments	`y_std_mean`	✓	✓
XY distribution / Moments	`y_std_mean_abs`	✓	✓
XY distribution / PCA	`eccentricity`	✓	✓
XY distribution / PCA	`radial`	✓	✓

1.2.1 Distribution along X and Y axes

Each neuronal reconstruction consisted of points with Euclidean coordinates, with the center of gravity of the soma located at coordinates $(0, 0, 0)$. Thus, computing, e.g., the standard deviation along the X axis provided an estimate of arborization extent in the horizontal direction.

1.2.1.1 PCA-derived

Following Yelnik et al. (1983) we used principal component analysis (PCA) to quantify possible preferential orientation of an arbor along either the X or Y dimension. We set the Z coordinates to zero and quantified such preference with the index of axialization measure of Yelnik et al. (1983), calling it `eccentricity`:

$$\mathrm{e} = 1 - \frac{s_2}{s_1},$$

where s_1 and s_2 are standard deviations of the first and second principal components, respectively (thus, $s_1 \geq s_2 \geq 0$). An e towards 1 indicates a strong preference for one axis, whereas an e towards 0 indicates a circular arbor. We used the angle θ of the main axis (i.e., the first principal component) to a positive X axis passing through the center of mass to quantify the degree of `radial` or tangential orientation of the arbor, namely,

$$\mathrm{r} = (|y| - |x|) \times \mathrm{e},$$

where y and x are the loadings of the first component on the Y and X axes, respectively, and correspond to $|\sin\theta|$ and $|\cos\theta|$. Thus, r is positive if the tree is ascending or descending, and close to -1 if it mainly arborizes horizontally. To reduce its magnitude for trees that did not have a preference for one of the two directions, we factored in the degree of eccentricity, e (which is always positive).

1.2.1.2 Moments along the axes (distribution around the soma)

We computed the mean, standard deviation, and the standardized mean (i.e., the ratio of the mean to the standard deviation) along the X and Y axes. The sign of `y_mean` and `y_std_mean`, for example, may help distinguish between arbors that ascend towards the pial surface or descend towards the white matter; unlike `y_mean`, `y_std_mean` is dimensionless and expresses the arborization preference in terms of the Y extent of the arbor. We also computed the means of $|x|$ and $|y|$ so as to not distinguish between arbors skewed towards the right or the left (or above or below) of the soma, but instead between those arborizing close and far from soma, both horizontally and vertically. The standard deviations indicate the extent along an axis and are very correlated with the `height` and `width` morphometrics. Finally, we computed the ratio of the range along an axis and the standard deviation along that axis (`ratio_x` and `ratio_y`).

1.2.1.3 Grid analysis

We split the X and Y plane into $20\,\mu m$ by $20\,\mu m$ squares, and computed the number of branches in each square. We recorded the number of non-empty squares (i.e., those containing at least one branch; `grid_area`), as an estimate of the arbor's area, and the mean (`grid_mean`) branch count per non-empty square. Finally, we computed the ratio of non-empty $100\,\mu m$ by $100\,\mu m$ squares and `grid_area`, to quantify arborization density (`grid_density`), i.e., arbors that tend to occupy a large of portion of a given $100\,\mu m$ by $100\,\mu m$ square.

1.2.1.4 Axon origin

In order to distinguish axons that originate from below the soma from those that originate above it, we recorded the Y coordinate of the first axonal bifurcation (`axon_origin`), as well as the difference between the minimal path distance from the soma among points more than $100\,\mu m$ below the soma ($Y < 100\ \mu m$) and those at least $100\,\mu m$ above the soma ($Y > 100\ \mu m$; `axon_above_below`); a positive value would suggest that the arborization begun on the upper side of the soma.

1.2.2 Laminar distribution

Since we did not know the exact location of the soma within a layer, we could only estimate axonal projection across the layers. For these estimates we relied on layer thickness data from Figure 3 of Markram et al. (2015), shown in Table 3, assuming that the thickness T_l of layer l follows a Gaussian distribution, $\mathcal{N}(mt_l, st_l)$, where mt_l and st_l are the mean and standard deviation of T_l (given in Table 3).

The probability of an axon reaching L1 depends on axonal height above the soma, h_a, and the distance D from the soma to the center of L1, c_1. We modelled D as a sum of two independent random variables, $D = D_l + P$, where D_l is the distance from c_l, the center of the soma's layer l, to c_1, and P the position of the soma with respect to c_l (considering, in both cases, only the Y dimension). Assuming layers' thicknesses are independent, $D_l \sim \mathcal{N}(md_l, sd_l)$, where

$$md_l = \frac{mt_1}{2} + \sum_{k=2}^{l-1} mt_k + \frac{mt_l}{2},$$

and

$$sd_l = \sqrt{\frac{st_1^2}{4} + \sum_{k=2}^{l-1} st_k^2 + \frac{st_l^2}{4}},$$

where the summation term is omitted for L2 (i.e., $l = 2$). Assuming that P follows $\mathcal{N}(0, \frac{mt_l}{4})$, the sum $D_l + P$ follows $\mathcal{N}(md_l, \sqrt{sd_l^2 + (\frac{mt_l}{4})^2})$ and the probability `l1_prob` of an axon reaching L1 is that of drawing a value equal or greater than h_a from this distribution. Thus, for example, for an L4 cell with its axon extending $500\,\mu m$ above its soma, the probability of reaching L1 was 0.0005, whereas for one with length $700\,\mu m$ was 0.6450, i.e., 65% ($md_4 = 679.5$; see Table 3).

Table 3: Layer thickness data from Markram et al. (2015) and the estimated distance from the layer's center to the center of L1.

Layer	Thickness	Distance to L1 ($md_l \pm sd_l$)
1	165 ± 13	
2/3	502 ± 27	333.5 ± 15
4	190 ± 7	679.5 ± 28
5	525 ± 33	1037.0 ± 33.1
6	700 ± 48	1649.5 ± 49.9

We estimated the probability p_a of an arbor extending into the layer above as the probability of drawing h_a from a Gaussian distribution $\mathcal{N}(\frac{mt_l}{2}, \frac{mt_l}{4})$, where h_a is the arbor's height above the soma, and mt_l, as above, the mean thickness of the soma's layer l. We computed the probability p_b of reaching the layer below analogously, setting it to 0 for layer L6. The probability of an arbor being `translaminar` (i.e., not confined to a single layer) was given by $\max\{p_a, p_b\}$.

1.2.3 MC arborization pattern

To estimate axonal width in L1 (`l1 width`; MC cells' axons tend to spread out horizontally in this layer), we computed its width in the upper 165 μm (i.e., the thickness of L1) of its arborization and multiplied it with the probability of having reached L1 (`l1_prob`). In an analogous way we estimated the total number of bifurcations in L1 (as a proxy for total arbor extent in that layer). We also estimated the extent to which this arborization grew horizontally (`l1_gx`) and away from the soma (`l1_gxa`), following the assumption that the axon rises vertically approximately above the soma, and ramifies in both horizontal directions in layer L1. `l1_gx` is the sum of all segments' X-axis projections, whereas `l1_gxa` equals `l1_gx` minus the X-axis projections of all segments directed towards the soma (i.e., their initial X coordinate is further from the soma than their terminal coordinate).

1.2.4 ChC arborization pattern

Since ChC cells' axons have short vertical terminals (Markram et al., 2004; Somogyi, 1977), we counted the number of terminal branches with an extent along the Y axis $< 50\,\mu m$ (Somogyi (1977) reports ChC vertical terminals from $10\,\mu m$ to $50\,\mu m$ long) and at least twice as large as the extent along the X axis (`short_vertical_terminals`).

1.2.5 Arbor density

We quantified arbor density with a number of ratios involving arbor length as the denominator: the ratio of the number of bifurcations and arbor length (`density_bifs`), proportional to the inverse of branch length; the ratio of `area` and arbor length (`density_area`), and, finally, the ratio of average Euclidean distance and total length (`density_dist`).

1.2.6 Dendritic bipolarity

We quantified whether the dendrites stemmed from opposite ends of the soma and whether those ends are located along a radial (i.e., parallel to the Y) axis, as is the case with bipolar and bitufted dendrites. We did this by applying the above-described PCA-derived analysis to the dendrite insertion points on the soma's surface, after having replicated every insertion point once for each whole μm of the corresponding dendrite's length, so as to give more weight to insertion points of longer dendrites, and having set the Z coordinates to 0. A high `insert.eccentricity` thus indicated insertion points along an axis, rather than spread-out across the soma's surface, whereas a high `insert.radial` suggested that the axis was parallel to the Y axis. For cells with a single dendrite insertion point we set `insert.eccentricity` and `insert.radial` to 0.

1.2.7 Displaced dendritic arbor

To quantify whether the dendritic arbor was displaced (DeFelipe et al., 2013) from the axonal one, we averaged the distance to the closest axonal reconstruction point for each point of a dendritic arbor (`displaced`).

1.3 Implementation

NeuroSTR is available at https://github.com/ComputationalIntelligenceGroup/neurostr. The code for computing custom-implemented features is available at https://github.com/ComputationalIntelligenceGroup/neurostrplus.

2 Supervised classification

2.1 Overview

We considered eight separate classification scenarios, one for each interneuron class (including the basket 'superclass') versus all others joined together. Most of these classification tasks were highly imbalanced, with one class much scarcer than the other. For each task, we carried out the same learning procedure —univariate feature selection, under- and over-sampling, and classifier learning— and estimated its performance with cross-validation.

More precisely, we first standardized the predictors over the entire data set, i.e., prior to cross-validation. Cross-validation then split the data sample into k training and test subsets. We performed feature selection and data sampling on the training data alone, and separately for each training subset. For each training subset, we performed, in the following order: 1) feature selection; 2) data under- and over-sampling; and 3) classifier learning, where we considered a number of different learning algorithms. Steps 1 and 2 were optional, giving the four combinations considered: feature selection followed by classifier learning (without data sampling); feature selection followed by data sampling and then classifier learning; classifier induction without sampling or feature selection; and finally, classifier induction without feature selection but with data sampling. We evaluated the induced classifiers on the k test subsets.

Besides the performance of supervised classification, we looked at the results of feature selection. We ranked and selected features according to the Kruskal-Wallis (KW) test and random forest balanced variable importance (RF BVI), a variation of the well-known random forest variable importance metric, defined below.

2.2 Notation and terminology

We denote the vector of n predictor variables or features with $\mathbf{X} = (X_1, \ldots, X_n)$ and the class variable with C. Lowercase $\mathbf{x}$ and c each denote a single assignment to the predictors $\mathbf{X}$ and the class C, respectively. In our setting, $\mathbf{x} \in \mathbb{R}^n$ while $c \in \{c_0, c_1\}$, the positive and negative classes. We have a data set $\mathcal{D} = \{(\mathbf{x}^{(i)}, c^{(i)})\}_1^N$ consisting of N instances or data points (i.e., interneurons) $\mathbf{x}^{(i)}$ with their label (interneuron class) $c^{(i)}$. A classifier is a function $f \colon \mathbb{R}^n \to \mathcal{C}$. A learning algorithm produces f from a training set of observed values of $\mathbf{X}$ and C. We may use the terms classifier, learning algorithm, and model interchangeably.

2.3 Supervised classifiers

We applied a number of state-of-the-art classifier learning algorithms (Murphy, 2012; Hastie et al., 2009). They are all listed in Table 2 in the main text, along with the abbreviations that we will use to refer to them in the following sections. Below we briefly describe them.

2.3.1 CART

The CART algorithm (Breiman et al., 1984) produces a decision tree by recursively partitioning the training samples according to a single predictor at a time. For each node a of the tree, CART selects the splitting predictor

X_j, and its threshold value t, by minimizing 'class impurity' G,

$$\min_{j,t}\{G(\mathcal{D}^a_{jt}) + G(\mathcal{D}^a \setminus \mathcal{D}^a_{jt})\},$$

where $\mathcal{D}^a$ is a subset of $\mathcal{D}$ at node a, while $\mathcal{D}^a_{jt}$ and $\mathcal{D}^a \setminus \mathcal{D}^a_{jt}$ are the left and right splits, respectively, of $\mathcal{D}^a$ according to X_j and threshold t,

$$\mathcal{D}^a_{jt} = \{(\mathbf{x}^{(i)}, c^{(i)}) : x_j^{(i)} \leq t, \mathbf{x}^{(i)} \in \mathcal{D}^a\}.$$

One measure of impurity is the Gini criterion,

$$G(\mathcal{D}^a) = \sum_{l=1}^{k} P_{\mathcal{D}^a}(c_l)(1 - P_{\mathcal{D}^a}(c_l)),$$

where $P_{\mathcal{D}^a}$ is the empirical probability of class c_l in $\mathcal{D}^a$. Deep trees can overfit the data, and options for regulating complexity include $|\mathcal{D}^a|$, the minimum size of $\mathcal{D}^a$ required in order to attempt a split, and $|\mathcal{D}^l|$, the minimum size of some leaf node $\mathcal{D}^l$.

2.3.2 Random forest

A CART tree can overfit the training data. Besides pruning, another way to reduce variance is to use an ensemble of trees, such as the random forest classifier (RF; Breiman, 2001). One draws T bootstrap (Efron, 1979) samples (size N samples from $\mathcal{D}$ with replacement), and on each learns an unpruned CART tree. At each split, consider only $m \leq n$ randomly selected features. To make a prediction, choose the majority class among the T trees. Due to averaging over bootstrap samples, the RF is generally robust to overfitting.

2.3.3 AdaBoost

Like random forest, AdaBoost (Freund and Schapire, 1997) is an ensemble of classification trees. The first tree is trained in regular fashion, with all instances having the same weight. For each following tree, the weight of the instances misclassified by the previous tree is increased. This way even weak individual trees can be combined into an accurate ensemble. Parameters of the method include the depth d of the individual trees, a regularization parameter $s \in [0, 1]$, and the number of trees T.

2.3.4 Naive Bayes

The naive Bayes (Minsky, 1961) is a simple approximation to the joint probability distribution $P(C, \mathbf{X})$. It assumes that predictors are conditionally independent given the class and classifies an instance according to

$$c^* = \arg\max_c P(c|\mathbf{x}) \propto P(c) \prod_{j=1}^{n} p(x_j|c).$$

Here we assume that each $p(X_j \mid c)$ is a Gaussian probability density with mean $\mu_{j,c}$ and variance $\sigma^2_{j,c}$. Albeit a simple model, the naive Bayes often performs well, generally due to its low variance.

2.3.5 k-nearest neighbors

kNN (Fix and Hodges, 1951) classifies an instance $\mathbf{x}$ according to its nearest neighbors in feature space, by choosing the most common class label among them. The number of neighbors k is a parameter to the model, with a lower value reducing bias but increasing variance (a lower k fits the training data better). The neighbors are usually identified using a variant of the Minkowski distance, such as Euclidean distance. A common extension is to predict c^* by giving more importance to the points that are closer to the target point. Kernel functions are a common means of expressing such weight functions, with weights decreasing smoothly with distance from the target point $\mathbf{x}$ (see, e.g., Hechenbichler and Schliep, 2004).

2.3.6 Regularized logistic regression

According to the (binomial) logistic regression model (e.g., Hastie et al., 2009, Chapter 4), the log odds of a class c_0 and class c_1 are a linear function of $\mathbf{x}$:

$$\ln \frac{P(c_0 \mid \mathbf{x})}{P(c_1 \mid \mathbf{x})} = \beta_0 + \boldsymbol{\beta}^T \mathbf{x},$$

where β_0 and $\boldsymbol{\beta}$ are the model's coefficients. While the $\boldsymbol{\beta}$ can be fit by maximum likelihood estimation, regularizing the model by shrinking them can reduce variance. The lasso (Tibshirani, 1996) regularization finds the $\boldsymbol{\beta}$ by maximizing:

$$\max_{\beta_0, \boldsymbol{\beta}} \frac{1}{N} \sum_{i=1}^{N} \log P(c^{(i)} \mid \mathbf{x}^{(i)}) - \lambda \sum_{j=1}^{n} |\beta_j|,$$

where $P(c^{(i)} \mid \mathbf{x}^{(i)})$ is the probability, under the model, of $c^{(i)}$ given $\mathbf{x}^{(i)}$, while λ specifies the degree of penalty on the magnitude of the coefficients. The lasso tends to shrink some coefficients to zero, effectively selecting, for interpretation purposes, the non-zero coefficient variables (features with $\beta_j = 0$ are effectively omitted from the model). The $\boldsymbol{\beta}$ coefficients are straightforward to interpret: keeping all other predictors fixed, a unit increase in a standardized predictor X_j increases the log-odds of the positive class by β_j. Thus, the higher $|\beta_j|$, the more useful is X_j. For groups of correlated predictors, lasso tends to keep a single non-zero coefficient and shrink the rest to zero. Implementations such as the `glmnet` package (Friedman et al., 2010) can efficiently optimize λ according to the cross-validated estimate of a loss function such as classification error.

2.3.7 Single-layer neural network

A single-layer neural network (Bishop, 1995) models $P(c \mid \mathbf{x})$ as a linear combination of derived features, also called hidden neurons, each of which is, in turn, a linear combination of $\mathbf{x}$. With the number of derived features $h = 0$, the neural network corresponds to a linear model such as logistic regression. Increasing h makes the model more flexible, with a sufficiently high h allowing it to represent any piecewise continuous function. The model is trained with an algorithm that minimizes cross-entropy loss.

2.3.8 Support vector machine

The SVM (Boser et al., 1992; Cortes and Vapnik, 1995) finds the maximal margin hyperplane that separates the two classes. It uses kernel functions to project the data onto a higher dimensional space, where they are more likely to be linearly separable. It searches for a separating hyperplane, determined by a coefficient vector $\boldsymbol{\beta}$ and an intercept β_0, by finding

$$\min_{\beta_0, \boldsymbol{\beta}, \boldsymbol{\xi}} \frac{1}{2} \boldsymbol{\beta}^T \boldsymbol{\beta} + R \sum_{i=1}^{N} \xi^{(i)}$$

$$\text{subject to } \xi^{(i)} \geq 0,\ c^{(i)} \phi(\boldsymbol{\beta}^T \mathbf{x}^{(i)} + \beta_0) \geq 1 - \xi^{(i)},\ \forall i,$$

with $c^{(i)} \in \{-1, 1\}$, $\xi^{(i)} = 0$ if $\mathbf{x}^{(i)}$ is on the correct side of the hyperplane, and $R > 0$ is the complexity parameter, with larger values narrowing the margin and yielding less training set misclassifications, while ϕ maps $\mathbf{x}$ to a higher dimensional space. ϕ is given by a kernel function K such that $K(\mathbf{x}, \mathbf{x}') = \phi(\mathbf{x})^T \phi(\mathbf{x}')$. A common example is the radial basis function, $K(\mathbf{x}, \mathbf{x}') = \exp\left(-\gamma ||\mathbf{x} - \mathbf{x}'||^2\right)$, whose parameter $\gamma > 0$ indicates spread from the target instance $\mathbf{x}$.

2.3.9 Linear discriminant analysis

Like multinomial regression, the LDA (Fisher, 1936; Rao, 1948) is a linear classifier, with piecewise hyperplanar decision boundaries. It assumes $p(\mathbf{x} \mid c_l) = \mathcal{N}(\boldsymbol{\mu}_l, \boldsymbol{\Sigma})$, that is, multivariate normal class-conditional distributions, with an $\boldsymbol{\mu}_l$ mean vector for each class c_l and a shared covariance matrix $\boldsymbol{\Sigma}$, equal for both classes.

2.4 Undersampling and oversampling

Most classifiers implicitly optimize classification accuracy, and with high class imbalance, this can lead to a poor prediction of the minority class. A standard technique for reducing this bias towards the majority class is to undersample or oversample the training data (He and Garcia, 2009) in order to achieve a more balanced training set.

Oversampling augments the training set with instances of the minority class. The SMOTE (Chawla et al., 2002) method creates a synthetic instance of the minority class by randomly choosing a point on the line between some minority class instance $\mathbf{x}$ and one of its k nearest neighbors from the minority class. Undersampling, on the other hand, involves removing a number of instances from the majority class. We combined both under- and over-sampling (as in, e.g., Estabrooks et al., 2004), using random undersampling followed by SMOTE oversampling. Finally, one has to determine the number of instances to add to (remove from) the training set; albeit a balanced training set is usually desirable, many synthetic minority class instances can lead to overfitting, while losing many majority class instances can mean losing valuable information (He and Garcia, 2009).

2.5 Feature selection

In small-sample class-imbalance settings, univariate feature selection (Guyon et al., 2006) can improve predictive performance more than over- and under-sampling, at least for the SVM (Wasikowski and Chen, 2010). We used the Kruskal-Wallis hypothesis test to identify univariately useful features. While not a commonly used feature selection technique (some examples are Golugula et al. (2011); Christin et al. (2013)), we expect it to be relatively insensitive to class imbalance. In addition, as a statistical hypothesis test, it provides a straightforward cut-point to discern relevant predictors from irrelevant ones: the p-value and a significance level, α, most commonly set to 0.05. We also used a multivariate feature ranking based on RF-derived variable importance (see below).

2.5.1 Kruskal-Wallis test

The null hypothesis of the Kruskal-Wallis test (Kruskal and Wallis, 1952) is that the medians of k samples are the same. In our case, these samples correspond to the k different classes. It is a non-parametric procedure and as such it does not assume that the data follow a particular distribution. Its special case for $k = 2$ is the Mann-Whitney-Wilcoxon test (Wilcoxon, 1945; Mann and Whitney, 1947). The test statistic H_j, for some feature X_j, is

$$H_j = (N-1)\frac{\sum_{l=1}^{k} N_l(\bar{r}_{l\cdot} - \bar{r})^2}{\sum_{l=1}^{k}\sum_{i=1}^{N_l}(r_{li} - \bar{r})^2},$$

where r_{li} is the rank of i-th sample in class c_l, $\bar{r}_{l\cdot}$ is the average rank of samples in class c_l, $\bar{r}$ is the average rank, and N_l is the number of instances in class c_l. H_j asymptotically follows the χ^2 distribution and thus we compute the test's p-value as $P(\chi^2_{k-1} \geq H_j)$. With small N_l, the χ^2 approximation is less accurate and results in reduced test power (Sheskin, 2003). We adjusted the p-values (obtained with the χ^2 test) for multiple testing by using the false discovery rate procedure (Benjamini and Hochberg, 1995).

2.5.2 RF variable importance

Variable importance (VI) is given by the out-of-bag (OOB) accuracy of the trees in the forest. An OOB sample for a tree t consists of instances which were not in the bootstrap subsample from which t was learned. Let a_t be the

percentage of correct classifications in the OOB sample for tree t, and a_{ptj} the percentage of correct classifications after randomly permuting the values of X_j in the OOB sample. Then,

$$VI(X_j) = \frac{1}{T}\sum_{t=1}^{T}(a_{tj} - a_{ptj}),$$

where $a_{tj} = a_{ptj} = 0$ if X_j is not in tree t; otherwise $a_{tj} = a_t$; T, as mentioned in Section 2.3.2, is the number of trees in the ensemble. Alternatively, one can compute per-class VIs by measuring changes in class-specific accuracies.

VI can loosely be interpreted as the feature's effect on accuracy and it provides a ranking of the features (obtained in a multivariate way). Useful features will have positive values whereas useless ones will have VIs around or below zero. A drawback is that the VI ranking tends to favor correlated predictors, especially for low values of m (i.e., the number of features considered at each split; see Strobl et al., 2008). Because the ranking is stochastic, it is important to use enough trees for it to stabilize.

The above-described VI is less effective in imbalanced settings, as misclassifications due to imbalance can overcome those due to class label permutation. While Janitza et al. (2013) proposed a VI derived from the change in area under the ROC curve (Swets, 1988; Fawcett, 2006), rather than the change in accuracy, so as to balance both types of errors (i.e., false positives and false negatives), we did not use it as it is only implemented for the RF variant based on conditional inference trees (Hothorn et al., 2006). Instead, we used the arithmetic mean of the per-class VI values provided by the `randomForest` `R` package, and refer to this as the balanced VI (BVI)[1].

Finally, given a VI-based ranking, it is not straightforward to determine the cut-point that separates useful features from useless ones. While Breiman (2001) suggests a statistical test for the purpose, it has some undesirable statistical properties (i.e., its power increases with the number of trees and decreases with sample size) and is thus not recommended (Strobl and Zeileis, 2008)[2]. Alternatives include permutation tests (Wang et al., 2010; Altmann et al., 2010) and methods based on OOB accuracy of nested RF models, corresponding to different cut-points along the ranking (Svetnik et al., 2003; Díaz-Uriarte and De Andres, 2006; Genuer et al., 2010). We used a simple heuristic and selected only features with a BVI above 0.01.

2.6 Detecting mislabelled examples

Wrong class labels may arise due to issues such as lack of relevant information, subjectivity, or simple human mistakes (Frénay and Verleysen, 2014). We followed the approach by Brodley and Friedl (1999), considering cells misclassified by different models as possibly mislabelled. It is beneficial for the models to belong to different paradigms, such as nearest neighbors and decision trees, so that their biases differ. The misclassifications correspond to cross-validation rather than resubstitution.

2.7 Unsupervised preprocessing

We standardized all predictors to zero mean and unit variance. This gives equal weight to all predictors for the kNN classifier, and allows us to interpret the magnitude of the coefficients of the linear models, while it does not affect the remaining models.

2.8 Assessing performance

On imbalanced data sets, a model can be accurate by simply predicting the majority class. We thus complemented the accuracy metric with the F-measure (Baeza-Yates and Ribeiro-Neto, 1999) score:

$$\text{F-measure} = \frac{2 \cdot \text{TP}}{2 \cdot \text{TP} + \text{FN} + \text{FP}},$$

[1] The VIs of the majority class are expected to be lower (Janitza et al., 2013) and using a harmonic or geometric mean, instead of the arithmetic, would further decrease the estimate, obfuscating possible effects in the minority class.

[2] The test is based on a scaled version of VI, divided by its standard error. The scaled VI also increases with the number of trees. We used the un-scaled version.

where TP, FP, and FN are entries of the confusion matrix, namely the true positives, the false positives, and false negatives, respectively. The F-measure thus balances different aspects of positive class prediction, namely the true positives, false positive, and the false negatives. In our case, the positive class was always the class of interest, e.g., when classifying ChC versus all other types, the positive type was ChC. Except for BA, the positive class was always considered the minority class (i.e., there were more BA than non BA cells).

We estimated the accuracy and F-measure with cross-validation (CV). Because over- and under-sampling introduced stochasticity into the learning process, we repeated CV a number of times and averaged to get the final estimates. Note that, unlike for classification accuracy, one cannot get an unbiased estimate of F-measure by averaging over the k test samples (Forman and Scholz, 2010); thus, we computed the F-measure of a CV run from the full confusion matrix, obtained by aggregating the true labels and predictions from the k test folds. That is, the F-measure estimate for a run of cross-validation was not the average of k per-fold F-measure scores, but rather the single value computed from the aggregated confusion matrix.

2.9 Parameters

We set the classifiers' parameters (see Table 2 in main text) on the basis of available recommendations (Boulesteix et al., 2012; Hsu et al., 2003) or we used the defaults in the software implementation. For kNN we used $k = 5$, and, similarly, for CART we set $|D^l| = 5$; while this might be too coarse-grained for ChC, as there are at most six ChC cells per training set, we sought to avoid overly complex models (with a lower k). Note that the $m = \sqrt{n}$ parameter for RF was recomputed on every training set; thus, it was adjusted each time feature selection reduced n. For RF, we set $T = 2000$ and chose the standard value of $m = \sqrt{n}$.

For KW feature selection, we set the significance level $\alpha = 0.05$, whereas for RF BVI ranking we selected features with BVI ≥ 0.01 and kept $m = \sqrt{n}$, although it can yield a BVI ranking that prefers correlated features (Nicodemus et al., 2010), while we increased the number of trees, T, to 20000, as that produced stable BVI values (a higher T does not increase model variance nor presents any other drawback besides longer computation time).

For undersampling, we randomly removed up to a half of the majority class instances, keeping at least three majority class instances per each one of the minority class (thus, for the imbalance ratios minority:majority above (i.e., less pronounced than) 1:3 we did not undersample). More precisely, after undersampling there were $N_M^{(u)} = \max(\min(3N_m, N_M), 0.5N_M)$ majority class instances, where N_M is the number of samples from the majority class and N_m that of the samples from the minority class. We then run SMOTE on the undersampled data set, adding up to three synthetic instances per each minority class example; thus, after oversampling there were $N_m^{(o)} = \min(N_M^{(u)}, 3N_m)$ minority class examples. Therefore, for large imbalances (e.g., a ratio 1:10) most balancing was due to undersampling, potentially reducing imbalance down to a ratio of to 1:3, with SMOTE oversampling then further reducing the ratio towards 1:1.

We evaluated the learning procedures with stratified 10-fold cross-validation, except for ChC versus rest, where we used seven folds (in order to have at least one ChC instance in each test set). When sampling the training data, we repeated CV 10 times and averaged the results. When computing F-measure, we always considered the minority class as the positive one, except for BA versus rest, when we considered BA, the majority class, as the positive one. We looked for mislabelled cells by collected misclassifications over 30 runs of ten-fold cross validation. Tables 2 and 3 in the main text list all the parameters.

2.10 Implementation

We implemented all the data analysis and classification in the `R` programming language (R Core Team, 2015). We used the `mlr` (Bischl et al., 2015) for classifier learning and evaluation, feature selection, oversampling and undersampling, extending it to compute a global F-measure for an entire cross-validation run and adding the FDR p-value correction to the KW feature selection method. All code and data are available at https://github.com/ComputationalIntelligenceGroup/bbp-interneurons-classify.

3 Morphology quality

3.1 Reconstruction differences

We found that the cells differed in mean axonal segment length and that this difference could be related to the internal ids (e.g., C010600B1 and MTC070301B_IDC) of the cells. Out of the seven different initial letters of these ids, cells whose id begun with a letter C (88 of them) had shorter, as well as thicker, axonal segments than the remaining 131 cells (see Figure 5 in main text). As branch and total arbor length did not differ between the two groups, this meant that the C-prefixed cells had fewer long and thick segments per branch, whereas the non-C cells contained more short and thin ones, suggesting that they were simply reconstructed at a finer granularity. We found that the C-prefixed cells were deposited at Neuromorpho.org repository (Ascoli et al., 2007) earlier than the non-C ones, meaning they may have been reconstructed at an earlier stage.

More morphometrics, such as axonal remote tilt angle (`remote_tilt_angle.avg`), arbor depth (`depth`), and tortuosity (`tortuosity.avg`), also differed between the two groups, albeit with much less statistical significance (see Table 4) than thickness and segment length. We suspect that only some of these, such as possibly tortuosity (the non-C cells have lower tortuosity, i.e., they are less straight, which seems logical given that they are broken into more segments) had been affected by differences in branch reconstruction granularity; others might have differed due to others causes, such as different proportion of interneuron types in the two groups, or the different laminar distribution (see Figure 1).

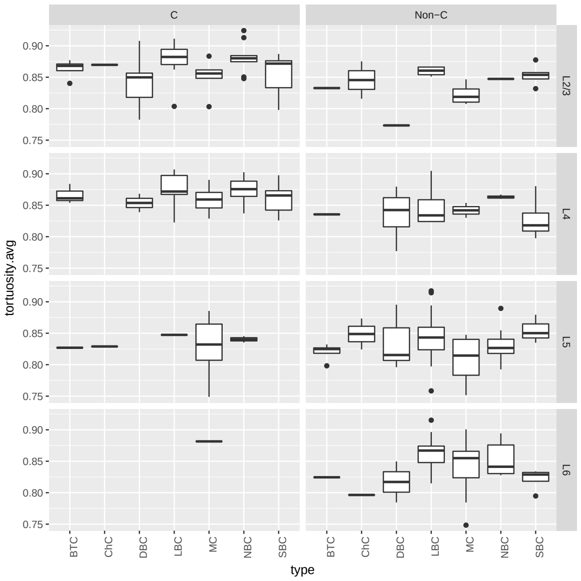

Figure 1: The branches of non-C cells' (those whose ids do not begin with a C) were less straight (i.e., their tortuosity values were lower), even after accounting for interneuron type and layer.

Table 4: Morphometrics that differed between the cells whose id begins with a C and the rest, according to a Kruskal-Wallis test at $\alpha = 0.05$, with the p-value corrected for multiple testing with the false discovery rate procedure (Benjamini and Hochberg, 1995).

Morphometric	Axon	Dendrite
compartment_length.avg	1.3×10^{-27}	
diameter.avg	5.3×10^{-26}	9.5×10^{-3}
N_nodes	6.2×10^{-22}	
remote_tilt_angle.avg	4.4×10^{-6}	2.6×10^{-2}
depth	2.5×10^{-5}	
tortuosity.avg	8.6×10^{-5}	
tortuosity.med	3.8×10^{-4}	8.9×10^{-3}
l1_gx	4.9×10^{-4}	
x_sd	1.4×10^{-3}	
density_area	3.2×10^{-3}	1.9×10^{-4}
l1_bifs	6.7×10^{-3}	
local_tilt_angle.avg	8.9×10^{-3}	
height	1.3×10^{-2}	8.0×10^{-4}
width	2.0×10^{-2}	2.0×10^{-2}
l1_gxa	2.1×10^{-2}	
euclidean_dist.max	2.3×10^{-2}	1.2×10^{-4}
y_mean	2.9×10^{-2}	
grid_area	3.8×10^{-2}	2.6×10^{-2}
density_dist		1.5×10^{-2}
euclidean_dist.avg		3.2×10^{-3}
euclidean_dist.sd		2.5×10^{-5}
path_dist.avg		3.8×10^{-2}
path_dist.max		1.6×10^{-3}
path_dist.sd		4.1×10^{-4}
ratio_x		2.6×10^{-2}
remote_torque_angle.avg		1.2×10^{-2}
terminal_degree.avg		1.6×10^{-3}
x_mean_abs		2.3×10^{-2}
y_sd		8.3×10^{-3}

3.2 Two cloned cells

We visually identified two cells as possible modified duplicates of another pair of cells (see Figure 2). They differed in most axonal and dendritic morphometrics, including the number of branches or axonal length, but were similar in axonal height and total dendritic length and height, suggesting how cells which are similar to the eye are not so according to most of the morphometrics that we are using. We then ran hierarchical clustering on all cells using these variables (i.e., axonal height, dendritic length and height) but found no additional pairs of duplicated cells.

4 Feature selection results

Table 5 shows the sizes of the feature subsets selected by the different methods and their performance.

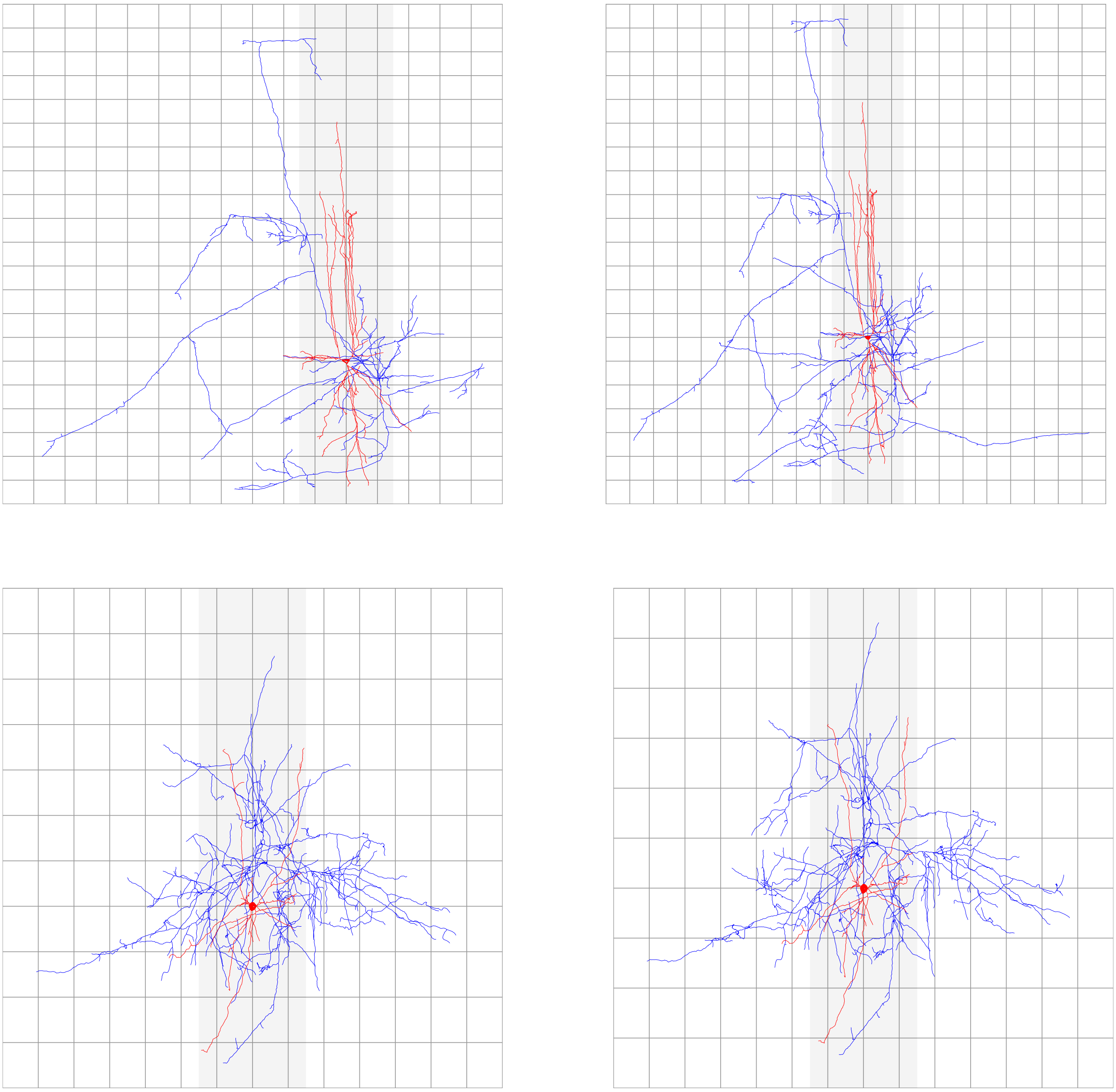

Figure 2: Cells OG061201A1-8_IDA and OG061201A3_CH1_IN_H_ZK_60X_1 (above), and OG061201A1-8_IDE and OG061201A6_CH5_BC_H_ZK_60X_1 (below) which seemed very similar by visual inspection. Axons are drawn in blue and dendrites and somata in red. There are 100 μm between consecutive grid lines.

Table 5: Number of selected morphometrics with the different methods. The color indicates the best F-measure obtained with the corresponding feature selection method. Best F-measure ≥ 0.75 are shown in green; best F-measure ≥ 0.60 in orange; and the rest in red. CART and RMLR refer to the embedded feature selection performed by those models. Filter feature selection followed by embedded selection is denoted with a +, e.g., KW followed by CART is denoted with KW + CART. CART and RMLR are only considered in absence of prior sampling. There are no entries for RF BVI + RMLR for the BTC as RMLR could not be fit due to too few features being selected by the RF BVI.

Class	KW	KW + CART	KW + RMLR	RF BVI	RF BVI + CART	RF BVI + RMLR	CART	RMLR
ChC	15	2	5	3	1	1	2	11
BTC	7	5	3	2	2		4	22
DBC	61	3	15	6	2	4	3	17
SBC	39	5	9	7	5	4	5	24
NBC	57	5	19	9	3	5	4	27
MC	62	6	22	8	5	6	5	28
LBC	32	9	17	4	4	4	8	38
BA	68	11	27	6	5	5	10	31

Table 6 shows the logistic regression model for MC.

Table 6: The logistic regression model for MC. The $\boldsymbol{\beta}$ were estimated from the standardized data set, after feature selection with KW.

Morphometric	β
`y_std_mean`	1.44
`ratio_y`	-0.88
`remote_bifurcation_angle.avg`	0.79
`path_dist.max`	0.63
`d.displaced`	-0.63
`l1_width`	0.59
`d.total_length`	0.47
`translaminar`	0.43
`radial`	0.31
`d.N_stems`	-0.30
`d.terminal_degree.avg`	-0.24
`density_bifs`	-0.23
`l1_bifs`	0.22
`d.path_dist.avg`	-0.22
`path_dist.avg`	0.14
`t.tortuosity.avg`	-0.14
`d.y_std_mean_abs`	0.13
`x_mean`	-0.11
`d.insert.radial`	-0.09
`t.length.med`	-0.09
`l1_prob`	0.01
`grid_density`	0.00

Table 7 shows the 88 features selected by KW for at least one of the types, showing the corresponding p-values. Each column corresponds to a one-versus-all classification setting. Overall, the single most relevant feature was `path_dist.avg` for BA, with a p-value of 3.6×10^{-17}, and the strongest dendritic predictor was the number of dendrites (`d.N_stems`) also for BA, with p-value 5.3×10^{-14}. Table 8 shows the features selected by RF BVI for the different classification settings, along with their RF BVI values. Overall, RF BVI selected only one dendritic morphometric, for the BTC type, and picked only axonal features for all remaining types.

Table 7: Morphometrics that differed most between the given class and the remaining classes joined together, according to the Kruskal-Wallis test. Empty entries mean that the p-value was above 0.05. Morphometrics that were significant for most classes are shown in the upper rows.

	Morphometric	ChC	BTC	DBC	SBC	NBC	MC	LBC	BA
1	`height`	3.8×10^{-3}		8.3×10^{-3}	2.4×10^{-7}	5.5×10^{-7}	4.2×10^{-6}	8.1×10^{-3}	2.7×10^{-7}
2	`d.displaced`	5.0×10^{-2}		8.5×10^{-4}		1.3×10^{-4}	2.2×10^{-4}	4.7×10^{-2}	3.0×10^{-10}
3	`d.insert.eccentricity`		4.3×10^{-2}	1.5×10^{-3}		8.6×10^{-4}	2.6×10^{-3}	4.5×10^{-2}	7.9×10^{-10}

4	euclidean_dist.max			3.6×10^{-3}	2.7×10^{-8}	1.1×10^{-10}	3.4×10^{-10}	2.0×10^{-2}	4.1×10^{-12}
5	grid_density	4.8×10^{-2}		3.1×10^{-4}	2.2×10^{-2}	1.7×10^{-4}	1.3×10^{-4}		1.6×10^{-8}
6	grid_mean	2.1×10^{-3}	1.2×10^{-2}		8.6×10^{-9}	2.0×10^{-2}	2.5×10^{-6}		3.2×10^{-7}
7	terminal_degree.avg	4.5×10^{-2}		7.3×10^{-3}		3.5×10^{-8}	2.0×10^{-7}	4.5×10^{-2}	2.7×10^{-9}
8	t.length.avg	3.8×10^{-3}	7.0×10^{-3}	7.3×10^{-3}	1.5×10^{-7}	2.4×10^{-3}		1.1×10^{-2}	
9	t.length.med			5.8×10^{-4}	4.3×10^{-3}	5.6×10^{-7}	1.8×10^{-3}	2.9×10^{-4}	2.1×10^{-8}
10	translaminar	5.5×10^{-3}		9.4×10^{-4}	1.2×10^{-5}	3.1×10^{-9}	7.0×10^{-8}		8.2×10^{-10}
11	y_sd	3.8×10^{-3}		1.1×10^{-3}	9.5×10^{-8}	6.3×10^{-9}	9.6×10^{-11}		2.5×10^{-12}
12	d.centrifugal_order.avg			2.7×10^{-3}		1.7×10^{-5}	5.6×10^{-7}	2.2×10^{-2}	3.1×10^{-13}
13	d.centrifugal_order.sd			1.0×10^{-2}	2.8×10^{-2}	7.0×10^{-4}	6.9×10^{-4}		7.9×10^{-8}
14	d.density_bifs			3.7×10^{-3}		1.4×10^{-5}	2.2×10^{-4}	2.8×10^{-3}	3.3×10^{-11}
15	density_area	3.8×10^{-3}			3.2×10^{-6}	3.4×10^{-3}	1.4×10^{-5}		1.5×10^{-6}
16	density_bifs	2.1×10^{-3}	2.4×10^{-3}		3.5×10^{-10}		1.4×10^{-2}	4.5×10^{-2}	
17	density_dist			8.2×10^{-7}		3.1×10^{-6}	5.5×10^{-7}	5.0×10^{-2}	3.6×10^{-13}
18	d.insert.radial		2.8×10^{-3}	7.7×10^{-4}		9.0×10^{-7}	2.8×10^{-2}		4.0×10^{-9}
19	d.N_bifurcations			3.8×10^{-2}		1.9×10^{-4}	1.3×10^{-7}	2.8×10^{-2}	1.8×10^{-11}
20	d.N_stems			6.9×10^{-4}		2.3×10^{-5}	5.0×10^{-6}	3.5×10^{-4}	5.3×10^{-14}
21	d.path_dist.avg			5.2×10^{-3}	2.6×10^{-2}	9.1×10^{-3}	1.3×10^{-2}		3.0×10^{-5}
22	d.terminal_degree.avg			1.4×10^{-3}		3.0×10^{-4}	1.1×10^{-3}	2.8×10^{-2}	3.4×10^{-9}
23	d.tree_length.avg			9.4×10^{-3}		8.1×10^{-4}	3.3×10^{-7}	1.3×10^{-2}	1.2×10^{-11}
24	euclidean_dist.avg			1.5×10^{-3}	6.1×10^{-9}	1.2×10^{-10}	1.2×10^{-13}		6.7×10^{-17}
25	euclidean_dist.sd			7.8×10^{-4}	6.1×10^{-9}	4.0×10^{-11}	7.1×10^{-13}		3.5×10^{-15}
26	length.avg	2.1×10^{-3}	2.4×10^{-3}		3.5×10^{-10}		1.4×10^{-2}	4.5×10^{-2}	
27	length.med	2.4×10^{-3}		1.6×10^{-2}	1.5×10^{-7}	1.1×10^{-2}		2.8×10^{-3}	
28	length.sd	2.1×10^{-3}	4.9×10^{-4}		3.5×10^{-10}		4.9×10^{-6}		2.4×10^{-6}
29	path_dist.avg			7.5×10^{-3}	3.2×10^{-6}	3.8×10^{-10}	6.8×10^{-14}		3.6×10^{-17}
30	path_dist.max			4.1×10^{-2}	5.5×10^{-7}	1.1×10^{-10}	4.0×10^{-13}		8.2×10^{-15}
31	path_dist.sd			1.3×10^{-3}	2.3×10^{-7}	1.2×10^{-10}	1.4×10^{-13}		6.7×10^{-17}
32	radial			4.6×10^{-8}	4.3×10^{-2}	3.1×10^{-6}	1.8×10^{-4}		9.8×10^{-9}
33	remote_bifurcation_angle.avg			3.1×10^{-4}		3.4×10^{-4}	1.2×10^{-5}	3.7×10^{-8}	7.8×10^{-15}
34	t.remote_bifurcation_angle.avg			1.3×10^{-4}		1.3×10^{-4}	5.2×10^{-6}	6.9×10^{-8}	3.4×10^{-15}
35	y_mean_abs			1.2×10^{-3}	8.3×10^{-4}	5.3×10^{-8}	1.0×10^{-10}		2.1×10^{-15}
36	y_std_mean_abs			1.7×10^{-2}		5.9×10^{-4}	1.7×10^{-4}	4.7×10^{-2}	1.6×10^{-8}
37	centrifugal_order.max			2.9×10^{-2}		1.3×10^{-5}	2.5×10^{-3}		5.5×10^{-4}
38	centrifugal_order.sd			7.7×10^{-4}		5.5×10^{-7}	3.9×10^{-5}		1.3×10^{-7}
39	d.centrifugal_order.max			3.1×10^{-3}		1.7×10^{-4}	2.9×10^{-5}		5.4×10^{-10}
40	d.euclidean_dist.avg			5.1×10^{-3}	1.2×10^{-2}	1.6×10^{-2}			6.0×10^{-4}
41	d.path_dist.max			9.4×10^{-3}	1.8×10^{-2}	4.0×10^{-2}			3.7×10^{-3}
42	d.y_sd			3.1×10^{-4}	1.2×10^{-2}	8.5×10^{-3}			7.2×10^{-3}
43	eccentricity			4.6×10^{-8}		7.2×10^{-8}	1.4×10^{-3}		5.3×10^{-8}
44	grid_area			1.3×10^{-4}	1.1×10^{-6}		1.4×10^{-3}	3.9×10^{-5}	
45	N_bifurcations	1.1×10^{-2}		5.8×10^{-4}	4.4×10^{-2}				1.2×10^{-2}
46	partition_asymmetry.avg			2.0×10^{-4}		8.2×10^{-7}	3.9×10^{-5}		1.9×10^{-9}
47	ratio_y			3.3×10^{-3}		4.4×10^{-6}	1.1×10^{-10}		5.0×10^{-12}
48	vertex_ratio			9.8×10^{-4}		5.5×10^{-6}	5.7×10^{-4}		1.9×10^{-7}
49	width			1.9×10^{-6}	2.1×10^{-4}		2.0×10^{-3}	3.8×10^{-3}	
50	x_sd			1.9×10^{-6}	3.4×10^{-4}		3.1×10^{-3}	4.6×10^{-3}	
51	y_mean			5.8×10^{-4}		9.1×10^{-3}	9.7×10^{-14}		1.4×10^{-3}
52	centrifugal_order.avg					3.5×10^{-5}	2.7×10^{-4}		1.9×10^{-4}
53	d.eccentricity			4.9×10^{-5}		2.5×10^{-3}			1.8×10^{-2}
54	d.euclidean_dist.max			5.6×10^{-3}	1.1×10^{-2}				1.6×10^{-2}
55	d.grid_area				4.0×10^{-3}		3.3×10^{-3}		3.1×10^{-2}
56	d.grid_mean						3.8×10^{-2}	1.3×10^{-2}	5.8×10^{-3}
57	d.height			1.1×10^{-3}	1.2×10^{-2}	3.1×10^{-2}			
58	d.length.avg					4.4×10^{-2}		7.3×10^{-3}	7.4×10^{-4}
59	d.path_dist.sd			3.4×10^{-2}	2.3×10^{-2}				2.8×10^{-2}
60	d.radial			8.2×10^{-7}		6.9×10^{-4}			1.2×10^{-3}
61	d.ratio_y			1.5×10^{-3}		2.7×10^{-2}			3.6×10^{-5}
62	l1_bifs					4.2×10^{-3}	1.2×10^{-7}		1.0×10^{-3}
63	l1_gx					2.2×10^{-2}	1.3×10^{-6}		2.4×10^{-3}
64	l1_gxa					8.7×10^{-4}	3.2×10^{-9}		4.5×10^{-5}
65	l1_prob					7.0×10^{-4}	2.2×10^{-8}		4.9×10^{-5}
66	l1_width					4.1×10^{-4}	1.0×10^{-10}		5.7×10^{-6}
67	remote_tilt_angle.avg				1.4×10^{-3}			3.4×10^{-6}	3.9×10^{-2}
68	short_vertical_terminals	2.7×10^{-3}		5.2×10^{-3}	1.3×10^{-3}				
69	tortuosity.avg						4.1×10^{-2}	2.7×10^{-2}	5.0×10^{-4}
70	t.remote_tilt_angle.avg				1.2×10^{-2}			4.6×10^{-6}	1.6×10^{-3}
71	t.tortuosity.avg						2.2×10^{-2}	4.5×10^{-2}	6.7×10^{-4}
72	y_std_mean			5.6×10^{-4}			1.6×10^{-10}		2.7×10^{-2}
73	axon_above_below					4.0×10^{-2}	1.7×10^{-2}		
74	axon_origin			1.6×10^{-2}			5.0×10^{-6}		
75	d.euclidean_dist.sd			2.4×10^{-2}	1.8×10^{-2}				
76	d.length.med					4.4×10^{-2}			1.3×10^{-3}
77	d.length.sd				3.6×10^{-2}			4.7×10^{-2}	
78	d.total_length				3.3×10^{-2}		1.4×10^{-3}		
79	d.y_mean_abs						4.1×10^{-2}		1.8×10^{-2}
80	tortuosity.med							5.0×10^{-2}	4.0×10^{-3}
81	total_length			6.5×10^{-6}				4.0×10^{-3}	
82	x_mean						5.6×10^{-4}		2.1×10^{-2}
83	d.density_dist			1.9×10^{-3}					
84	d.partition_asymmetry.avg								2.4×10^{-3}
85	d.translaminar			4.5×10^{-3}					
86	d.width			2.9×10^{-3}					
87	d.x_sd			8.5×10^{-4}					
88	d.y_std_mean_abs						1.6×10^{-2}		
	Dendritic	1	2	26	12	21	17	11	26
	Total	15	7	61	39	57	62	32	68

Table 8: Morphometrics that differed between the given class and the remaining classes joined together, according to the RF BVI ranking. Empty entries mean that the RF BVI for that class was above 0.01. Morphometrics that were relevant to most classes are shown in the upper rows.

	Morphometric	ChC	BTC	DBC	SBC	NBC	MC	LBC	BA
1	`density_bifs`	0.02			0.03	0.01			
2	`euclidean_dist.avg`				0.02	0.02			0.01
3	`euclidean_dist.sd`				0.02	0.04		0.02	
4	`length.avg`	0.02			0.03	0.01			
5	`path_dist.sd`					0.02	0.01		0.01
6	`euclidean_dist.max`					0.03		0.01	
7	`length.sd`	0.01			0.02				
8	`path_dist.avg`						0.02		0.02
9	`path_dist.max`					0.02	0.01		
10	`remote_bifurcation_angle.avg`							0.02	0.02
11	`t.length.avg`		0.01		0.01				
12	`t.remote_bifurcation_angle.avg`							0.02	0.02
13	`y_mean`			0.03			0.03		
14	`y_std_mean`			0.01			0.01		
15	`axon_origin`		0.01						
16	`d.insert.radial`		0.01						
17	`eccentricity`			0.04					
18	`l1_gxa`						0.01		
19	`l1_width`						0.01		
20	`length.med`				0.01				
21	`radial`			0.02					
22	`ratio_y`						0.01		
23	`translaminar`					0.01			
24	`width`			0.02					
25	`x_sd`			0.02					
26	`y_mean_abs`								0.01
27	`y_sd`					0.02			
	Dendritic	0	1	0	0	0	0	0	0
	Total	3	3	6	7	9	8	4	6

5 Classification results

Figures 3 to 10 show all classifiers' F-measure for all eight classification tasks. For ChC and BTC the results depended more strongly on sampling, with some samplings providing better and other worse results (e.g., for ChC, the F-measure of RF BVI + SVM ranged from 0.13 to 0.57; see Figure 3), as in these settings the amount of removed instances was highest. Perhaps an informed, rather than random, undersampling scheme could have improved the results.

5.1 Multi-class

We carried out multi-class classification by combining one-versus-all models. Figure 11 shows the accuracies of the different methods while Table 9 is the confusion matrix of the most accurate method, RF with KW feature selection and data sampling.

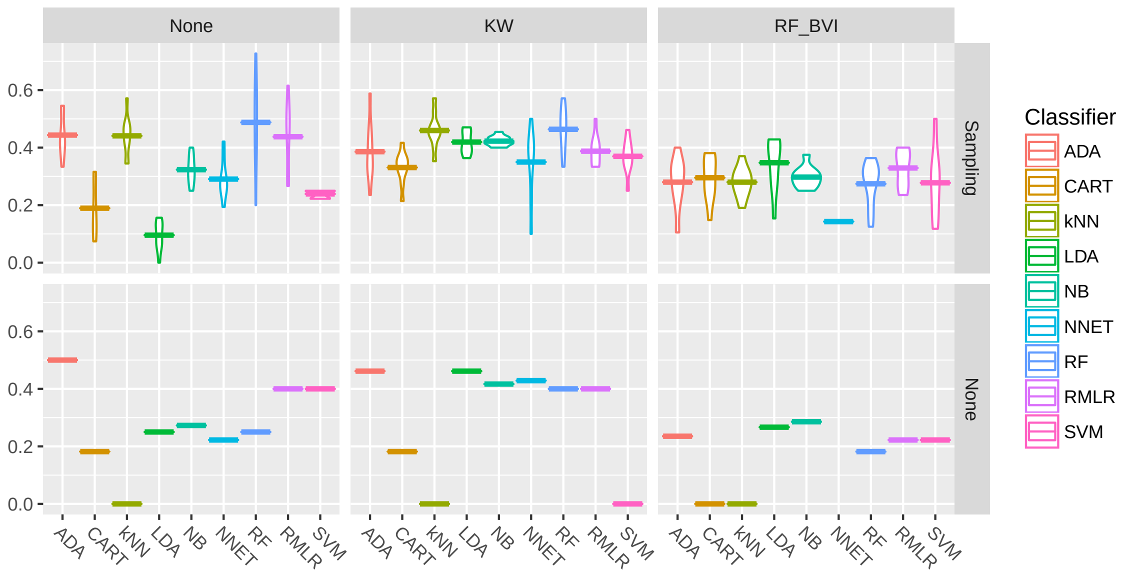

Figure 3: ChC versus rest. Violin plot of 7-fold cross-validation estimates of F-measure. Above: seven CV repetitions when under- and over-sampling training data; below: a single CV repetition with no data sampling. Vertical rows of panels correspond to the feature selection methods applied: none, KW, and RF BVI.

Table 9: Confusion matrix for a one-versus-all combination of the RF with KW feature selection and sampling. For each row, the positives are instances of the corresponding type whereas negatives are instances of all remaining types.

ChC	BTC	DBC	SBC	NBC	MC	LBC	F-measure	TPR	TNR
1	0	0	5	0	0	1	0.22	1 / 7	209 / 210
0	6	2	1	1	3	2	0.46	6 / 15	197 / 202
0	1	17	0	0	3	1	0.74	17 / 22	188 / 195
1	0	0	20	5	2	0	0.67	20 / 28	177 / 189
0	1	0	2	37	0	4	0.8	37 / 44	162 / 173
0	1	2	2	0	42	3	0.82	42 / 50	157 / 167
0	2	3	2	5	2	37	0.75	37 / 51	155 / 166

6 Neuroscientists' F-measure for the MC type

42 neuroscientists classified 320 cells in DeFelipe et al. (2013). For 299 those cells, at least 22 (half + one) of them agreed on single type, which we then considered as the true type of that interneuron; 48 of those cells were MC and 251 non-MC. We computed the F-measure of each neuroscientists with respect to the determined true type. The average F-measure was 0.72, minimal 0.12 and maximal 0.89, with only three neuroscientists performing better than our best MC model (F-measure 0.81).

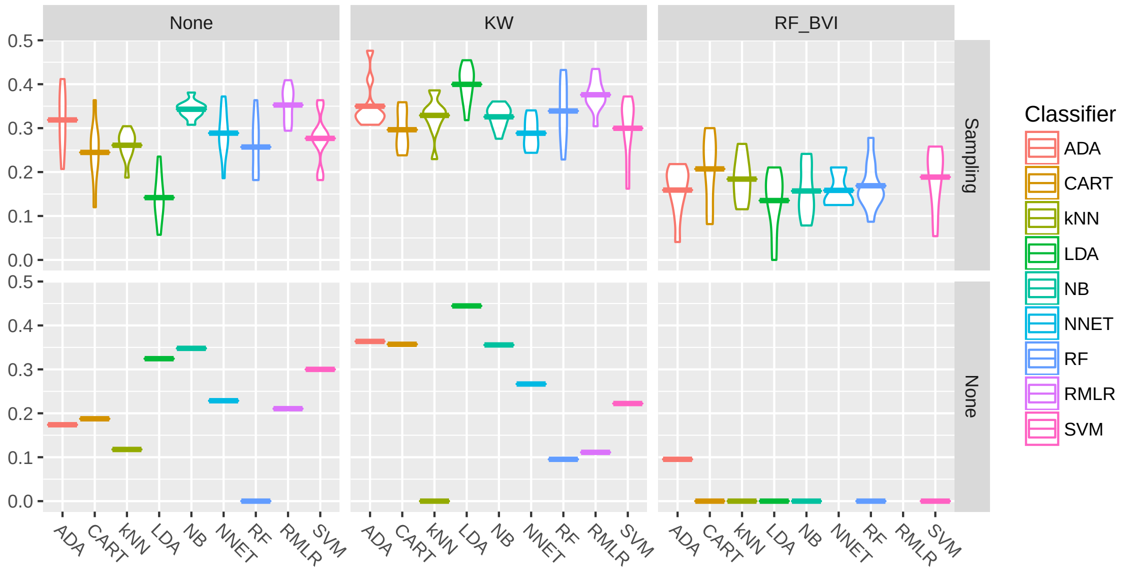

Figure 4: BTC versus rest. Violin plot of 10-fold cross-validation estimates of F-measure. Above: ten CV repetitions when under- and over-sampling training data; below: a single CV repetition with no data sampling. Vertical rows of panels correspond to the feature selection methods applied: none, KW, and RF BVI.

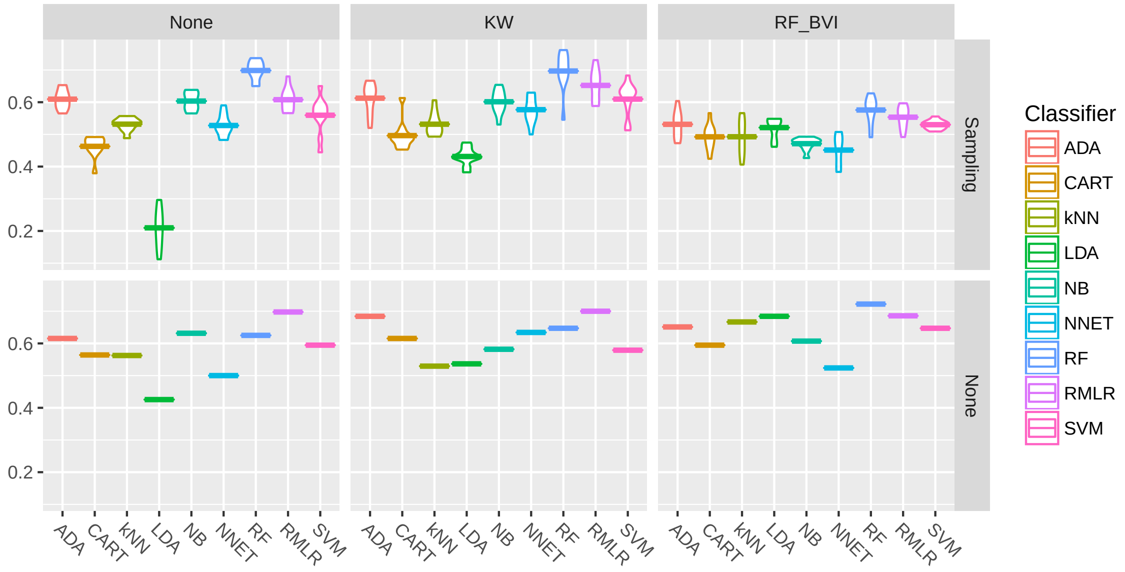

Figure 5: DBC versus rest. Violin plot of 10-fold cross-validation estimates of F-measure. Above: ten CV repetitions when under- and over-sampling training data; below: a single CV repetition with no data sampling. Vertical rows of panels correspond to the feature selection methods applied: none, KW, and RF BVI.

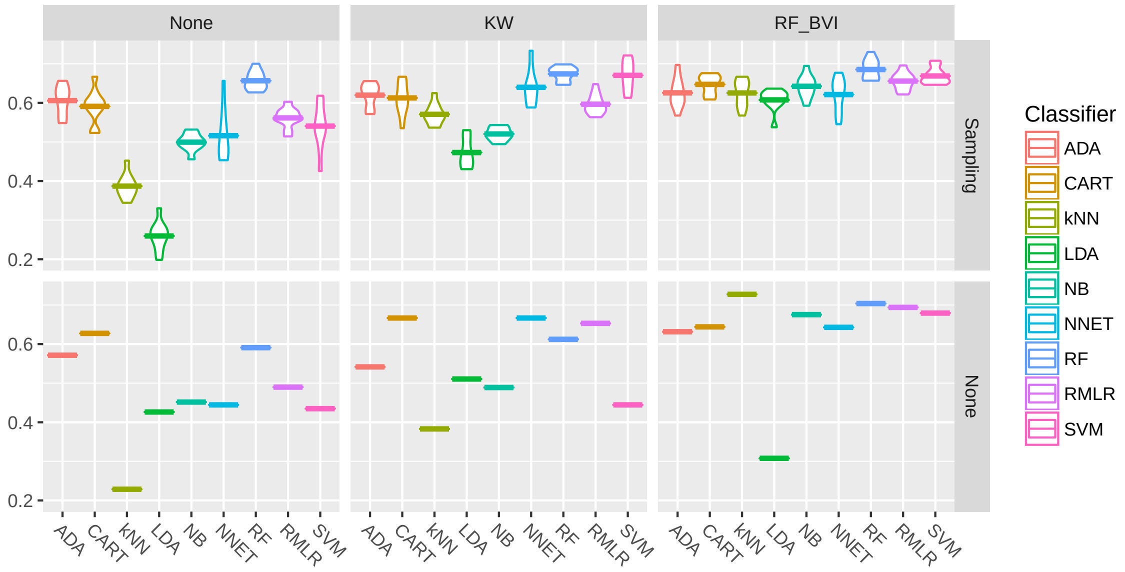

Figure 6: SBC versus rest. Violin plot of 10-fold cross-validation estimates of F-measure. Above: ten CV repetitions when under- and over-sampling training data; below: a single CV repetition with no data sampling. Vertical rows of panels correspond to the feature selection methods applied: none, KW, and RF BVI.

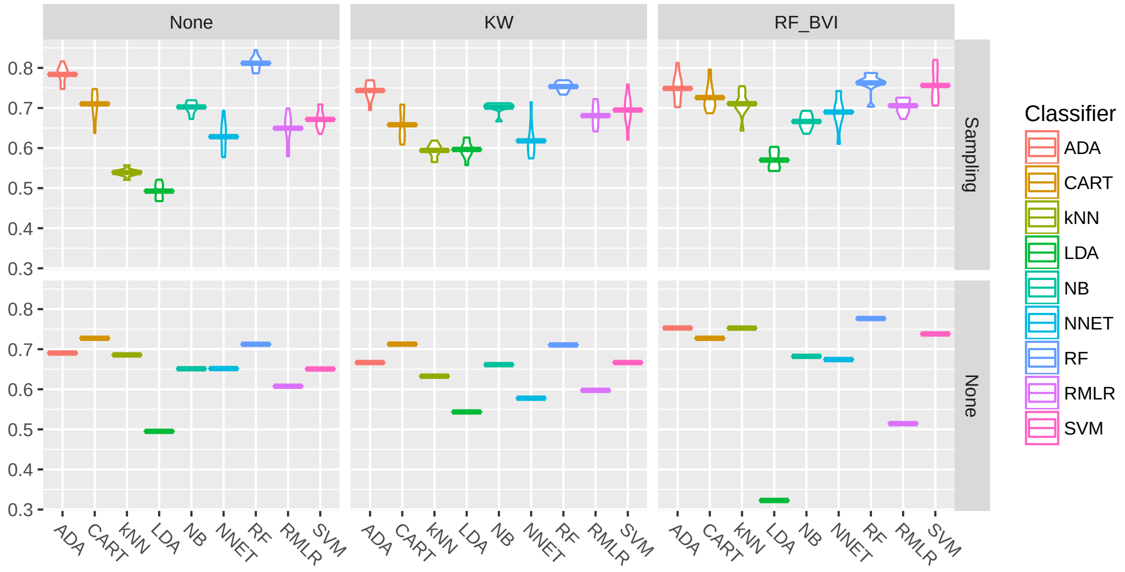

Figure 7: NBC versus rest. Violin plot of 10-fold cross-validation estimates of F-measure. Above: ten CV repetitions when under- and over-sampling training data; below: a single CV repetition with no data sampling. Vertical rows of panels correspond to the feature selection methods applied: none, KW, and RF BVI.

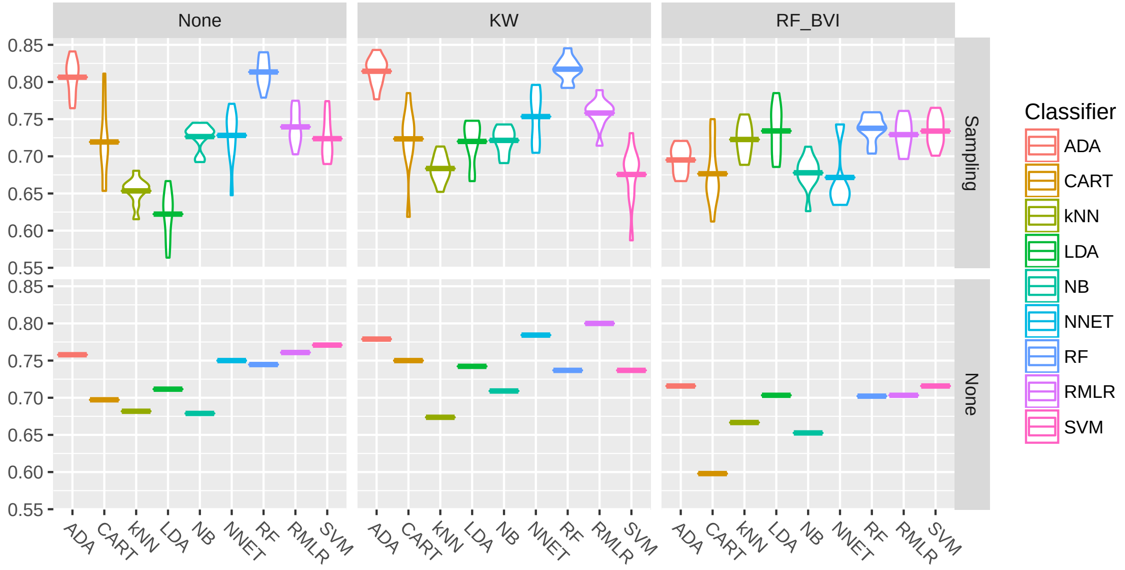

Figure 8: MC versus rest. Violin plot of 10-fold cross-validation estimates of F-measure. Above: ten CV repetitions when under- and over-sampling training data; below: a single CV repetition with no data sampling. Vertical rows of panels correspond to the feature selection methods applied: none, KW, and RF BVI.

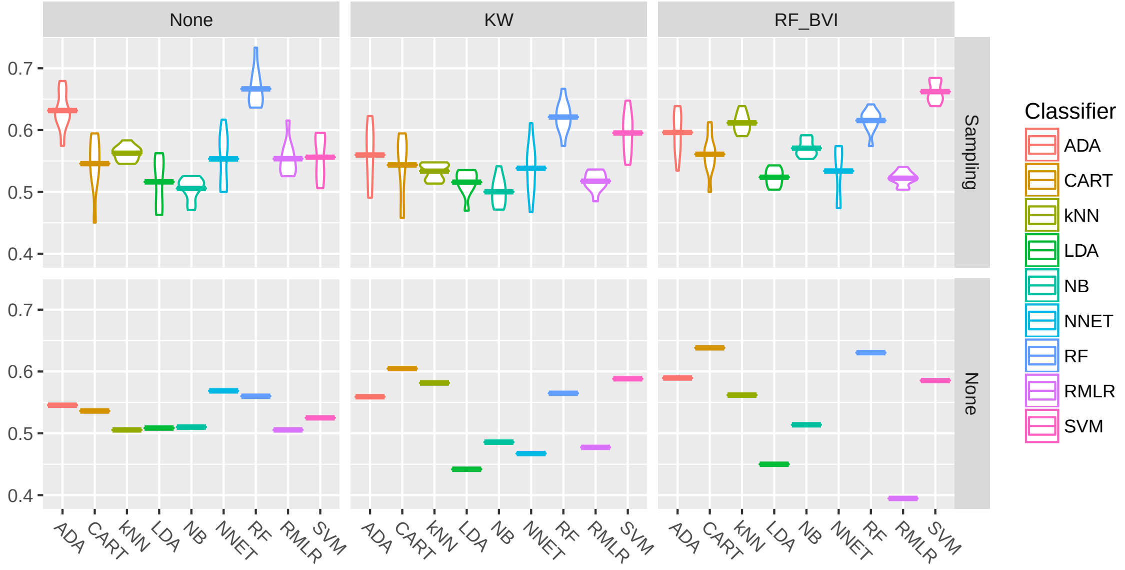

Figure 9: LBC versus rest. Violin plot of 10-fold cross-validation estimates of F-measure. Above: ten CV repetitions when under- and over-sampling training data; below: a single CV repetition with no data sampling. Vertical rows of panels correspond to the feature selection methods applied: none, KW, and RF BVI.

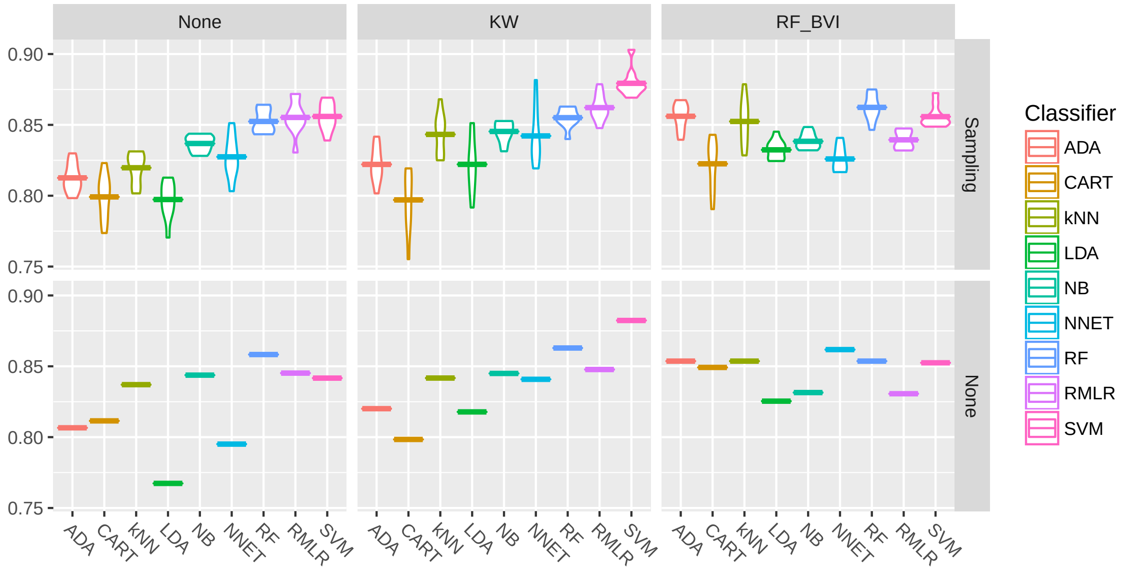

Figure 10: BA versus rest. Violin plot of 10-fold cross-validation estimates of F-measure. Above: ten CV repetitions when under- and over-sampling training data; below: a single CV repetition with no data sampling. Vertical rows of panels correspond to the feature selection methods applied: none, KW, and RF BVI. KW feature selection improved the performance of multiple models, most notably kNN, LDA, and SVM.

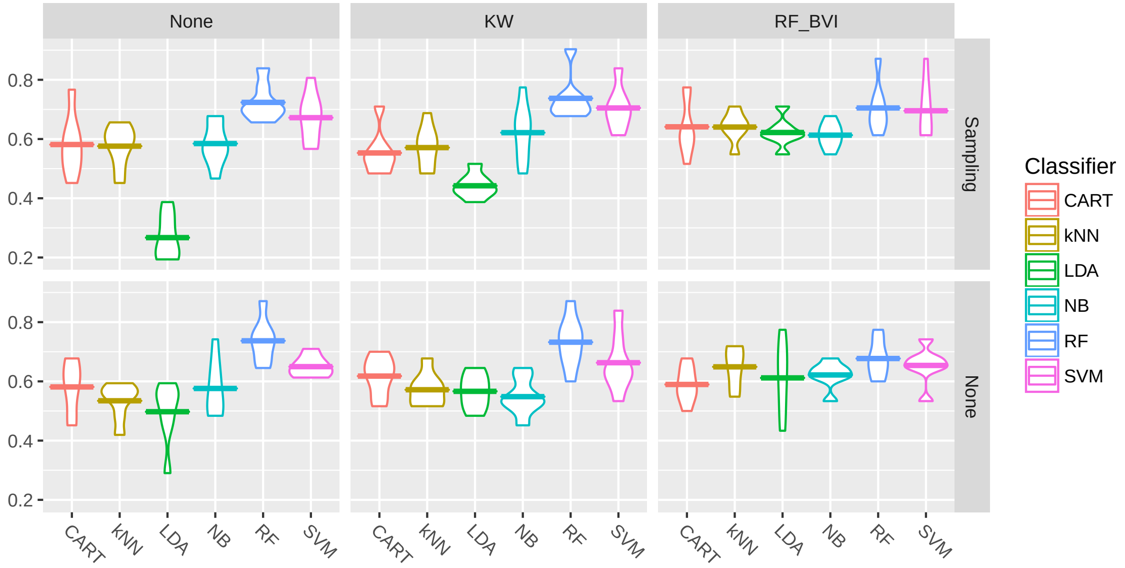

Figure 11: Multi-class classification by combining one-versus all models. Violin plot of 7-fold cross-validation estimates of F-measure. Above: ten CV repetitions when under- and over-sampling training data; below: a single CV repetition with no data sampling. Vertical rows of panels correspond to the feature selection methods applied: none, KW, and RF BVI. Entries are missing for ADA, NNET, and RMLR as their execution did not complete.

References

Altmann, A., Toloşi, L., Sander, O., and Lengauer, T. (2010). Permutation importance: A corrected feature importance measure. *Bioinformatics*, 26(10):1340–1347.

Ascoli, G. A., Donohue, D. E., and Halavi, M. (2007). Neuromorpho.org: A central resource for neuronal morphologies. *The Journal of Neuroscience*, 27(35):9247–9251.

Baeza-Yates, R. A. and Ribeiro-Neto, B. (1999). *Modern Information Retrieval*. Addison-Wesley Longman Publishing Co., Inc., Boston, MA, USA.

Benjamini, Y. and Hochberg, Y. (1995). Controlling the false discovery rate: A practical and powerful approach to multiple testing. *Journal of the Royal Statistical Society. Series B (Methodological)*, 57(1):289–300.

Bischl, B., Lang, M., Richter, J., Bossek, J., Judt, L., Kuehn, T., Studerus, E., and Kotthoff, L. (2015). *mlr: Machine Learning in R*. R package version 2.4.

Bishop, C. M. (1995). *Neural Networks for Pattern Recognition*. Oxford University Press.

Boser, B. E., Guyon, I. M., and Vapnik, V. N. (1992). A training algorithm for optimal margin classifiers. In *Proceedings of the Fifth Annual Workshop on Computational Learning Theory*, pages 144–152. ACM.

Boulesteix, A.-L., Janitza, S., Kruppa, J., and König, I. R. (2012). Overview of random forest methodology and practical guidance with emphasis on computational biology and bioinformatics. *Wiley Interdisciplinary Reviews: Data Mining and Knowledge Discovery*, 2(6):493–507.

Breiman, L. (2001). Random forests. *Machine Learning*, 45(1):5–32.

Breiman, L., Friedman, J., Stone, C. J., and Olshen, R. A. (1984). *Classification and Regression Trees*. Wadsworth, New York, NY, USA.

Brodley, C. E. and Friedl, M. A. (1999). Identifying mislabeled training data. *Journal of Artificial Intelligence Research*, 11:131–167.

Chawla, N. V., Bowyer, K. W., Hall, L. O., and Kegelmeyer, W. P. (2002). SMOTE: Synthetic minority over-sampling technique. *Journal of Artificial Intelligence Research*, 16:321–357.

Christin, C., Hoefsloot, H. C., Smilde, A. K., Hoekman, B., Suits, F., Bischoff, R., and Horvatovich, P. (2013). A critical assessment of feature selection methods for biomarker discovery in clinical proteomics. *Molecular & Cellular Proteomics*, 12(1):263–276.

Cortes, C. and Vapnik, V. (1995). Support-vector networks. *Machine Learning*, 20(3):273–297.

DeFelipe, J., López-Cruz, P. L., Benavides-Piccione, R., Bielza, C., Larrañaga, P., Anderson, S., Burkhalter, A., Cauli, B., Fairén, A., Feldmeyer, D., Fishell, G., Fitzpatrick, D., Freund, T. F., González-Burgos, G., Hestrin, S., Hill, S., Hof, P. R., Huang, J., Jones, E. G., Kawaguchi, Y., Kisvárday, Z., Kubota, Y., Lewis, D. A., Marín, O., Markram, H., McBain, C. J., Meyer, H. S., Monyer, H., Nelson, S. B., Rockland, K., Rossier, J., Rubenstein, J. L. R., Rudy, B., Scanziani, M., Shepherd, G. M., Sherwood, C. C., Staiger, J. F., Tamás, G., Thomson, A., Wang, Y., Yuste, R., and Ascoli, G. A. (2013). New insights into the classification and nomenclature of cortical GABAergic interneurons. *Nature Reviews Neuroscience*, 14(3):202–216.

Díaz-Uriarte, R. and De Andres, S. A. (2006). Gene selection and classification of microarray data using random forest. *BMC Bioinformatics*, 7(1):3.

Efron, B. (1979). Bootstrap methods: Another look at the jackknife. *The Annals of Statistics*, 7(1):1–26.

Estabrooks, A., Jo, T., and Japkowicz, N. (2004). A multiple resampling method for learning from imbalanced data sets. *Computational Intelligence*, 20(1):18–36.

Fawcett, T. (2006). An introduction to ROC analysis. *Pattern Recognition Letters*, 27(8):861–874.

Fisher, R. A. (1936). The use of multiple measurements in taxonomic problems. *Annals of Human Genetics*, 7(2):179–188.

Fix, E. and Hodges, J. L. (1951). Discriminatory analysis-nonparametric discrimination: Consistency properties. Technical Report 4, USAF School of Aviation Medicine, Randolph Field.

Forman, G. and Scholz, M. (2010). Apples-to-apples in cross-validation studies: Pitfalls in classifier performance measurement. *ACM SIGKDD Explorations Newsletter*, 12(1):49–57.

Frénay, B. and Verleysen, M. (2014). Classification in the presence of label noise: A survey. *IEEE Transactions on Neural Networks and Learning Systems*, 25(5):845–869.

Freund, Y. and Schapire, R. E. (1997). A decision-theoretic generalization of on-line learning and an application to boosting. *Journal of Computer and System Sciences*, 55(1):119–139.

Friedman, J., Hastie, T., and Tibshirani, R. (2010). Regularization paths for generalized linear models via coordinate descent. *Journal of Statistical Software*, 33(1):1–22.

Genuer, R., Poggi, J.-M., and Tuleau-Malot, C. (2010). Variable selection using random forests. *Pattern Recognition Letters*, 31(14):2225–2236.

Golugula, A., Lee, G., and Madabhushi, A. (2011). Evaluating feature selection strategies for high dimensional, small sample size datasets. In *2011 Annual International Conference of the IEEE Engineering in Medicine and Biology Society*, pages 949–952.

Guyon, I., Gunn, S., Nikravesh, M., and Zadeh, L. (2006). *Feature Extraction: Foundations and Applications.* Springer-Verlag, Berlin, Germany.

Hastie, T. J., Tibshirani, R. J., and Friedman, J. H. (2009). *The Elements of Statistical Learning: Data Mining, Inference, and Prediction.* Springer Series in Statistics. Springer, New York, NY, USA.

He, H. and Garcia, E. A. (2009). Learning from imbalanced data. *IEEE Transactions on Knowledge and Data Engineering*, 21(9):1263–1284.

Hechenbichler, K. and Schliep, K. (2004). Weighted k-nearest-neighbor techniques and ordinal classification. Technical Report Discussion paper 399, SFB 386, Ludwig-Maximilians University, Munich.

Hothorn, T., Hornik, K., and Zeileis, A. (2006). Unbiased recursive partitioning: A conditional inference framework. *Journal of Computational and Graphical Statistics*, 15(3):651–674.

Hsu, C.-W., Chang, C.-C., and Lin (2003). A practical guide to support vector classification. Technical report, Department of Computer Science and Information Engineering, National Taiwan University.

Janitza, S., Strobl, C., and Boulesteix, A.-L. (2013). An AUC-based permutation variable importance measure for random forests. *BMC Bioinformatics*, 14(1):119.

Kruskal, W. H. and Wallis, W. A. (1952). Use of ranks in one-criterion variance analysis. *Journal of the American Statistical Association*, 47(260):583–621.

Mann, H. B. and Whitney, D. R. (1947). On a test of whether one of two random variables is stochastically larger than the other. *The Annals of Mathematical Statistics*, (1):50–60.

Markram, H., Muller, E., Ramaswamy, S., Reimann, M., Abdellah, M., Sanchez, C., Ailamaki, A., Alonso-Nanclares, L., Antille, N., Arsever, S., Kahou, G., Berger, T., Bilgili, A., Buncic, N., Chalimourda, A., Chindemi, G., Courcol, J.-D., Delalondre, F., Delattre, V., Druckmann, S., Dumusc, R., Dynes, J., Eilemann, S., Gal, E., Gevaert, M., Ghobril, J.-P., Gidon, A., Graham, J., Gupta, A., Haenel, V., Hay, E., Heinis, T., Hernando, J., Hines, M., Kanari, L., Keller, D., Kenyon, J., Khazen, G., Kim, Y., King, J., Kisvarday, Z., Kumbhar, P., Lasserre, S., Le Bé, J.-V., Magalhães, B., Merchán-Pérez, A., Meystre, J., Morrice, B., Muller, J., Muñoz-Céspedes, A., Muralidhar, S., Muthurasa, K., Nachbaur, D., Newton, T., Nolte, M., Ovcharenko, A., Palacios, J., Pastor, L., Perin, R., Ranjan, R., Riachi, I., Rodríguez, J.-R., Riquelme, J., Rössert, C., Sfyrakis, K., Shi, Y., Shillcock, J., Silberberg, G., Silva, R., Tauheed, F., Telefont, M., Toledo-Rodriguez, M., Tränkler, T., Van Geit, W., Díaz, J., Walker, R., Wang, Y., Zaninetta, S., DeFelipe, J., Hill, S., Segev, I., and Schürmann, F. (2015). Reconstruction and simulation of neocortical microcircuitry. *Cell*, 163(2):456 – 492.

Markram, H., Toledo-Rodriguez, M., Wang, Y., Gupta, A., Silberberg, G., and Wu, C. (2004). Interneurons of the neocortical inhibitory system. *Nature Reviews Neuroscience*, 5(10):793–807.

Minsky, M. (1961). Steps toward artificial intelligence. *Transactions on Institute of Radio Engineers*, 49:8–30.

Murphy, K. P. (2012). *Machine Learning: A Probabilistic Perspective.* The MIT Press, Cambridge, MA, USA.

Nicodemus, K. K., Malley, J. D., Strobl, C., and Ziegler, A. (2010). The behaviour of random forest permutation-based variable importance measures under predictor correlation. *BMC Bioinformatics*, 11(1):110.

R Core Team (2015). *R: A Language and Environment for Statistical Computing.* R Foundation for Statistical Computing, Vienna, Austria.

Rao, C. R. (1948). The utilization of multiple measurements in problems of biological classification. *Journal of the Royal Statistical Society. Series B (Methodological)*, 10(2):159–203.

Sheskin, D. J. (2003). *Handbook of Parametric and Nonparametric Statistical Procedures.* CRC Press.

Somogyi, P. (1977). A specific 'axo-axonal' interneuron in the visual cortex of the rat. *Brain Research*, 136(2):345–350.

Strobl, C., Boulesteix, A.-L., Kneib, T., Augustin, T., and Zeileis, A. (2008). Conditional variable importance for random forests. *BMC Bioinformatics*, 9(1):307.

Strobl, C. and Zeileis, A. (2008). Danger: High power!–exploring the statistical properties of a test for random forest variable importance. In *Proceedings of the 8th International Conference on Computational Statistics, Porto, Portugal.*

Svetnik, V., Liaw, A., Tong, C., Culberson, J. C., Sheridan, R. P., and Feuston, B. P. (2003). Random forest: A classification and regression tool for compound classification and QSAR modeling. *Journal of Chemical Information and Computer Sciences*, 43(6):1947–1958.

Swets, J. A. (1988). Measuring the accuracy of diagnostic systems. *Science*, 240(4857):1285–1293.

Tibshirani, R. (1996). Regression shrinkage and selection via the lasso. *Journal of the Royal Statistical Society. Series B (Methodological)*, 58(1):267–288.

Wang, M., Chen, X., and Zhang, H. (2010). Maximal conditional chi-square importance in random forests. *Bioinformatics*, 26(6):831–837.

Wasikowski, M. and Chen, X. (2010). Combating the small sample class imbalance problem using feature selection. *IEEE Transactions on Knowledge and Data Engineering*, 22(10):1388–1400.

Wilcoxon, F. (1945). Individual comparisons by ranking methods. *Biometrics Bulletin*, 1(6):80–83.

Yelnik, J., Percheron, G., Francois, C., and Burnod, Y. (1983). Principal component analysis: A suitable method for the 3-dimensional study of the shape, dimensions and orientation of dendritic arborizations. *Journal of Neuroscience Methods*, 9(2):115–125.
